# Structural and evolutionary insights into astacin metallopeptidases

**DOI:** 10.3389/fmolb.2022.1080836

**Published:** 2023-01-04

**Authors:** F. Xavier Gomis-Rüth, Walter Stöcker

**Affiliations:** ^1^ Proteolysis Laboratory, Department of Structural Biology, Molecular Biology Institute of Barcelona (IBMB), Higher Scientific Research Council (CSIC), Barcelona, Catalonia, Spain; ^2^ Institute of Molecular Physiology (IMP), Johannes Gutenberg-University Mainz (JGU), Mainz, Germany

**Keywords:** evolution of metallopeptidases, catalytic domain (CD), darwinian descent, horizontal gene transfer (HGT), phylogeny of enzymes

## Abstract

The astacins are a family of metallopeptidases (MPs) that has been extensively described from animals. They are multidomain extracellular proteins, which have a conserved core architecture encompassing a signal peptide for secretion, a prodomain or prosegment and a zinc-dependent catalytic domain (CD). This constellation is found in the archetypal name-giving digestive enzyme astacin from the European crayfish *Astacus astacus*. Astacin catalytic domains span ∼200 residues and consist of two subdomains that flank an extended active-site cleft. They share several structural elements including a long zinc-binding consensus sequence (HEXXHXXGXXH) immediately followed by an EXXRXDRD motif, which features a family-specific glutamate. In addition, a downstream SIMHY-motif encompasses a “Met-turn” methionine and a zinc-binding tyrosine. The overall architecture and some structural features of astacin catalytic domains match those of other more distantly related MPs, which together constitute the metzincin clan of metallopeptidases. We further analysed the structures of PRO-, MAM, TRAF, CUB and EGF-like domains, and described their essential molecular determinants. In addition, we investigated the distribution of astacins across kingdoms and their phylogenetic origin. Through extensive sequence searches we found astacin CDs in > 25,000 sequences down the tree of life from humans beyond Metazoa, including Choanoflagellata, Filasterea and Ichtyosporea. We also found < 400 sequences scattered across non-holozoan eukaryotes including some fungi and one virus, as well as in selected taxa of archaea and bacteria that are pathogens or colonizers of animal hosts, but not in plants. Overall, we propose that astacins originate in the root of Holozoa consistent with Darwinian descent and that the latter genes might be the result of horizontal gene transfer from holozoan donors.

## 1 Introduction

The astacins are a family of extracellular zinc-dependent metallopeptidases (MPs) named after a digestive enzyme discovered in the 1960s in the European crayfish *Astacus astacus* L., as named by Linnaeus (Linnaeus, 1758), which was also referred to as *Astacus fluviatilis* F. by Fabricius (Fabricius, 1796). The enzyme was first named “*Astacus* protease” or “low-molecular-weight protease” (Pfleiderer et al., 1967), and the designation “astacin” was coined after related proteins had been found in other organisms [for reviews, see (Dumermuth et al., 1991; Jiang and Bond, 1992; Stöcker et al., 1993; Bond and Beynon, 1995; Zwilling and Stöcker, 1997; Gomis-Rüth et al., 2012a; Stöcker et al., 2013a; Stöcker et al., 2013b; Bond, 2019)]. Moreover, the astacins were the first identified members of the metzincin clan of MPs together with the matrix metalloproteinases, serralysins and adamalysins/a-disintegrin-and-metalloproteinases (ADAMs), which share common topologies and zinc-binding environments as inferred from structural studies (Bode et al., 1993; Stöcker and Bode, 1995; Gomis-Rüth, 2003; Sterchi, 2008; Gomis-Rüth, 2009; Cerdà-Costa and Gomis-Rüth, 2014; Arolas et al., 2018). Astacins function as protein degraders during digestion, developmental tissue turnover and differentiation, and embryonic hatching, but also as sophisticated shedders of membrane-bound substrates (Jiang and Bond, 1992; Gomis-Rüth et al., 2012a; Stöcker et al., 2013b; Bond, 2019). They are subdivided into the bone morphogenetic protein 1 (BMP1)/“Tolloid”-like proteinases (BTPs), meprins, hatching enzymes, and other astacins (Sterchi et al., 2008). Recent genomes have unravelled a plethora of genes encoding proteins annotated as astacins in various organisms within metazoans, which date back to 760 million years ago (Berman 2019).

In this article, we both dissected reported molecular structures and calculated new high-confidence computational models to analyse the molecular determinants of the most relevant astacin domains. Based on structural and molecular specifications of the prototypic astacin catalytic domain (CD), we further performed comprehensive sequence similarity searches to identify potential family members outside vertebrates to locate the origin of astacins according to Darwinian descent. Finally, we screened and reviewed the literature available for functional and evolutionary implications of the distinct astacin subfamilies outside vertebrates.

## 2 Results and discussion

### 2.1 Architecture and function of relevant astacin-family domains

**FIGURE 1:**
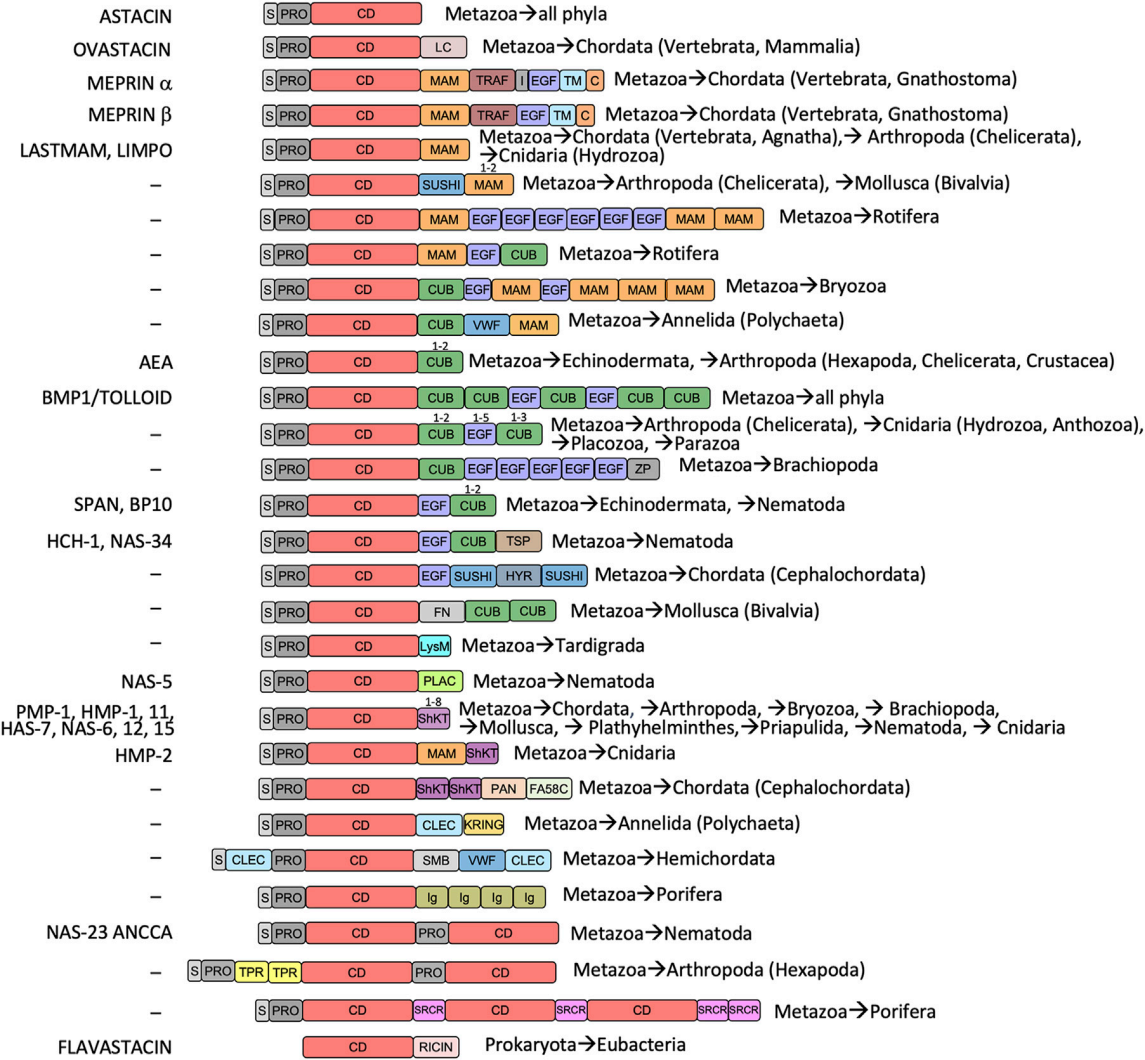
Astacin domain combinations and phylogenetic occurrence. Metazoan astacins minimally comprise an N-terminal signal-peptide for extracellular secretion (S), a propeptide or prodomain conferring latency (PRO) and a catalytic zinc-dependent metallopeptidase domain (CD). Moreover, most astacins evince additional domains, listed with *Prosite* database codes (PS; https://prosite.expasy.org): ABC (ABC-transporter; PS00211), CD (catalytic protease domain; PS51864), C (cytoplasmic tail), CLEC (C-type lectin; PS50041), CUB (found in complement subcomponents C1r/C1s, Uegf and BMP1; PS01180), EGF (epidermal growth factor-like; PS00022), FA58C (factor-5/8 type-C domain; PS500229), FN3 (fibronectin type-III domain; PS508539), HYR (hyalin repeat protein; PS50825), I (intervening domain in meprin α, contains a furin cleavage site), IG (immunoglobulin-like; PS508359), KRING (kringle; PS00021), LC (low complexity domain, disordered), LCCL (*Limulus* C-domain; PS50820), LYSM (extracellular receptor domain; PS51782), MAM (found in meprin, A5 protein and receptor protein tyrosine phosphatase μ; PS00740), MATH (meprin and traf homology domain; PS50144), PAN (also dubbed APPLE; found in plasma kallikrein and factor XI; PS50948), PLAC (polycystin-1, lipoxygenase and α-toxin; PS50095), PTX (pentraxin; PS51828), RICIN (ricin-type lectin; PS50231), SH2 (SARC homology domain; PS50001), ShKT (K^+^-channel-blocking *Stichodactyla helianthus* toxin; PS51670), SMB (somatomedin; PS50958), SRCR (cysteine-rich scavenger receptor; PS50287), SUSHI (sushi adhesion domain; PS50923), TPR (tetratricopeptide repeat; PS50005), TSP (thrombospondin-like domain; PS50092), VWF (von-Willebrand-factor domain; PS50234), ZF-UBR (UBR-type zinc-finger; PS51157) and ZP2 (zona pellucida protein 2 domain; PS51034). On the left, typical astacin family members are listed, for which physiological functions are documented (see Supplementary Table S1 for complete protein and gene names, and *UniProt* access codes). Phylogenetic occurrences are indicated on the right.

Astacins across all phyla minimally comprise a zinc-binding CD, which is preceded by an upstream propeptide or prodomain (PRO) for latency and a signal peptide (S) for targeting to the plasmalemma or the extracellular space in animals [(Gomis-Rüth et al., 2012a); Figure 1]. However, most astacins are multidomain proteins, which have acquired a diverse set of additional domains (Figure 1). We retrieved reported experimental crystal structures of a CD, a “MAM” domain [first identified in meprin, A5 protein and receptor protein tyrosine phosphatase μ; (Cismasiu et al., 2004)], a “TRAF” domain [reminiscent of tumour-necrosis-factor receptor-associated factor; (Park, 2018)] and PRO domains, and computed high-confidence computational models of the “CUB” domain [first identified at the sequence level in complement subcomponents C1r/C1s, Uegf and BMP1; (Bork and Beckmann, 1993)] and epidermal growth factor (EGF)-like domains (see the Methods section) for their molecular analysis.

**FIGURE 2:**
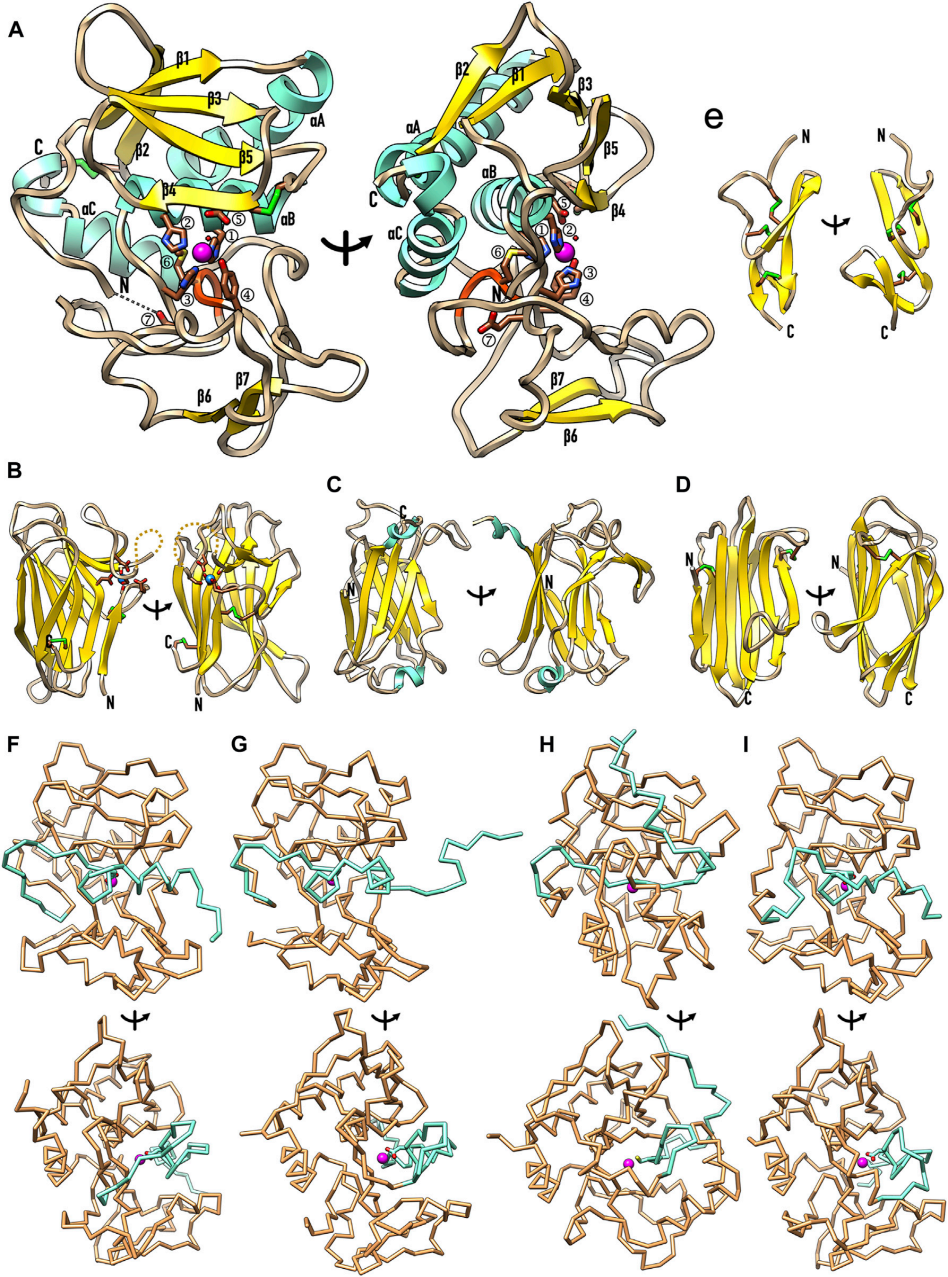
Representative structures of the most relevant astacin domains. **(A)** Ribbon-type plot of the mature *Astacus astacus* crayfish astacin catalytic domain [PDB 1AST; residues 50–251, see *UniProt* P07584; (Bode et al., 1992; Gomis-Rüth et al., 1993)], which is shown in the standard orientation of MPs [left; (Gomis-Rüth et al., 2012b)] and vertically rotated by 90 degrees (right). Regular secondary structure elements are shown as yellow β-strands (β1–β7) and aquamarine α-helices (αA–αC). The first five strands constitute the typical five-stranded β-sheet of astacins (Gomis-Rüth et al., 2012a) and the helices are dubbed “backing helix” (αA), “active-site helix” (αB) and “C-terminal helix” (αC). The latter is split in two by a kink. Unbound mature astacin has its catalytic zinc cation (magenta sphere) bound in trigonal-bipyramidal coordination by the three histidines (①–③) of a characteristic zinc-binding motif [HEXXHXXGXXH; (Bode et al., 1993)] plus a more distal downstream tyrosine (④) and the catalytic solvent molecule [small red sphere; (Arolas et al., 2018)]. The glutamate within the motif (⑤) is the general base/acid for catalysis (Arolas et al., 2018). The “Met-turn” with the conserved methionine [⑥; (Bode et al., 1993; Tallant et al., 2010)] is shown as an orange ribbon. The mature N-terminal residue (labelled N) is bound to the family-specific glutamate (E^103^) [⑦; (Gomis-Rüth, 2003)] after the third zinc-binding histidine. The C-terminus is also labelled (C) and the two disulfide bonds of the structure (C^42^–C^198^ and C^64^–C^84^) are further displayed with sulphur atoms in green. **(B)** The structure of the unique EGF-like domain of human BMP1 predicted with *AlphaFold* (Jumper et al., 2021) shows two β-ribbons and three disulfide bonds. Two orthogonal orientations are displayed. **(C)** Experimental structure of the MAM domain of meprin β [PDB 4GWM; (Arolas et al., 2012)] in two orthogonal orientations. The β-sandwich domain (residues 259–427, see *UniProt* Q16820) features two disulfide bonds and a structural sodium cation (blue sphere) octahedrally coordinated by six protein oxygens. **(D)** Structure of the first CUB domain of human BMP predicted with *AlphaFold* in two orthogonal orientations, which show a β-sandwich architecture with two disulfide bonds. **(E)** Experimental structure of the TRAF domain of meprin β [PDB 4GWM; (Arolas et al., 2012)] in two orthogonal orientations. The β-sandwich domain (residues 428–597, see *UniProt* Q16820) has two short helices and a β-ribbon grafted into strand-connecting loops. **(F–I)** Experimental zymogen structures as Cα–traces in standard orientation (top panels) and after a vertical 90-degree rotation (bottom panels) of **(F)** crayfish astacin [PDB 3LQ0; (Guevara et al., 2010)], **(G)** human meprin β [PDB 4GWM; (Arolas et al., 2012)], **(H)** myroilysin from the bacterium *Myroides* sp. [PDB 5GWD; (Xu et al., 2017)] and **(I)** astacin from the horseshoe crab *Limulus polyphemus* [PDB 8A28; (Guevara et al., 2022)]. Only the PROs (aquamarine) and CDs (sandy brown) are displayed for clarity, together with the catalytic zinc ions (magenta spheres) and the side chains of the respective aspartate/cysteine-switch residue.

The CD is ascribed to protein family Pfam-01400 (Figure 1) and spans ∼200 residues. It contains two or three disulfide bonds at variable positions (Gomis-Rüth et al., 2012a) and is divided into an upper N-terminal subdomain and a lower C-terminal subdomain by an extended active-site cleft, as first revealed by the crystal structure of archetypal crayfish astacin (Bode et al., 1992; Gomis-Rüth et al., 1993) (Figure 2A). The N-terminal subdomain is rich in regular secondary structure and contains a hallmark five-stranded β-sheet (β1–β5; Figure 2A), whose lowermost strand β4 frames the upper rim of the active-site cleft when viewed in the standard orientation of MPs (Gomis-Rüth et al., 2012b) (Figure 2A, left), and two helices: the “backing helix” and the “active-site helix”. The latter encompasses most of a characteristic zinc-binding motif (HEXXHXXGXXH; amino-acid one letter code; X stands for any residue), which is found across astacins and other metzincin families (Bode et al., 1993; Stöcker et al., 1993; Yiallouros et al., 2000; Gomis-Rüth et al., 2012a; Cerdà-Costa and Gomis-Rüth, 2014; Arolas et al., 2018). The helix includes the first two zinc-liganding histidines and the general base/acid glutamate required for catalysis (Arolas et al., 2018). After the glycine of the motif, the chain undergoes a sharp turn and enters the C-terminal subdomain, which is more irregular and just encompasses a short β-ribbon (β6–β7) and a “C-terminal helix” as regular secondary structure (Figure 2A). The C-terminal subdomain provides two more zinc ligands, viz., the third histidine of the motif and a downstream tyrosine, which is swung out in a “tyrosine-switch” motion upon substrate binding to stabilize the reaction intermediate during catalysis (Grams et al., 1996; Yiallouros et al., 2000). This tyrosine is found two positions after another conserved element within metzincins, the “Met-turn” methionine (Bode et al., 1993; Tallant et al., 2010), which creates a hydrophobic base for the metal-binding site (Tallant et al., 2010). The tyrosine and the methionine are embedded in a characteristic SIMHY-motif in astacins (Stöcker et al., 1993).

Finally, another structural characteristic of astacin CDs is an unaccessible N-terminus (Gomis-Rüth et al., 2012a; Guevara et al., 2022). Maturation cleavage occurs at a bond that is occluded in the zymogen, which entails that partial unfolding of the segment flanking the activation site and/or preliminary cleavages are required for activation (Guevara et al., 2010; Arolas et al., 2012; Guevara et al., 2022). Upon final cleavage, the first six or seven residues of the mature enzyme are amply repositioned and penetrate the mature enzyme moiety, so the first two or three residues are completely buried in the molecular structure (Gomis-Rüth et al., 1993; Arolas et al., 2012; Ran et al., 2020; Guevara et al., 2022). Moreover, the new N-terminus binds the “family-specific” glutamate immediately after the third zinc-binding histidine (Bode et al., 1993; Gomis-Rüth, 2003), either directly through its side chain or through the α-amino group *via* a solvent molecule (Figure 2A). This feature is unique among MPs and reminiscent of trypsin-like serine endopeptidases, which dedicate an aspartate next to the catalytic serine to bind the likewise buried mature N-terminus (Bode et al., 1986). The astacin glutamate is immediately followed by an XXRXDRD motif (Gomis-Rüth, 2003) whose charged residues establish interactions relevant for domain stability.

A MAM domain is found after the CD in meprins α and β, *Limulus* and *Hydra* astacins and other (potential) family members (Figure 1) (Arolas et al., 2012; Eckhard et al., 2021; Guevara et al., 2022). The crystal structure of human meprin β (Arolas et al., 2012; Eckhard et al., 2021) reveals that its MAM domain is a β-sandwich consisting of a four- and a five-stranded antiparallel β-sheet, which are twisted and rotated ∼25 degrees relative to each other (Figure 2B). The domain conforms to a jelly-roll architecture featuring two four-stranded Greek key motifs and is connected by two disulfide bonds. Furthermore, the domain has a sodium-binding site, at which the cation is octahedrally coordinated by six oxygens from side chains and the main chain of the protein (Figure 2B). The overall architecture of the domain conforms to the structural criteria defined for the MAM protein family (Pfam-00629), which was identified *in silico* in meprin *α* and *β*, A5 protein, and receptor protein tyrosine phosphatase μ (Beckmann and Bork, 1993). Comparison with other MAM domains reveals that the central β-sandwich is conserved but the loops responsible for functionality deviate, as well as the metal-binding capacity and arrangement (Aricescu et al., 2006; Yelland and Djordjevic, 2016). This domain appears to have adhesive functions and, in meprin *β*, it contributes to dimerization by bringing the CD and TRAF domains together (Arolas et al., 2012; Eckhard et al., 2021).

Uniquely for astacins, meprins α and β exhibit a TRAF domain downstream of the MAM domain (Figure 1) (Arolas et al., 2012; Eckhard et al., 2021). The crystal structure of human meprin β (Arolas et al., 2012; Eckhard et al., 2021) shows that this moiety features two twisted four-stranded antiparallel β-sheets, which are rotated ∼40 degrees relative to each other (Figure 2C, left) and give rise to a flatter sandwich than in MAM (compare Figure 2B, right and Figure 2C, right). The strands are connected by loops of variable length, which include two short helical segments plus a short β-ribbon and give rise to a double Greek key architecture. The second Greek key is inserted into the first one but does not form a jelly roll. The only cysteine of this domain (C^492^) is buried and unbound, the N- and the C-terminus are on contiguous β-strands of the front β-sheet (Figure 2C, left), the C-terminus protrudes from the top surface of the domain (Figure 2C, right). In general, the TRAF domain of meprin β resembles tumor-necrosis-factor receptor-associated factors, which are mediators of cell activation engaged in homo- and heterodimerization and originated the TRAF protein family (Pfam-00917) (Zapata et al., 2001).

Further relevant for astacins are CUB domains (Figure 1), which were first identified in complement subcomponents C1r/C1s, Uegf and BMP1 (Bork and Beckmann, 1993) and form protein family Pfam-00431. They occur in BTP-subfamily astacins including BMP1, as well as in echinoderm astacins, a paralogue within *A. astacus* and several other orthologues in up to five copies (Figure 1). According to a highly reliable *AlphaFold* computational model (see Figure 2D and the Methods section), the first CUB domain of human BMP1 would be a β-sandwich made of an antiparallel four-stranded β-sheet and a mixed parallel/antiparallel five-stranded β-sheet, which would be both partially twisted. Their strands would be nearly parallel (Figure 2D, left), in contrast to MAM (Figure 2B, left) and TRAF (Figure 2C, left), and connected by mostly short loops. Two disulfide bonds would crosslink the domain. CUB domains were apparently present in the last common ancestor of eumetazoans and are currently found in synaptic proteins (González-Calvo et al., 2022). Remarkably, combinations of CUB and MAM domains are found in neuropilins, which are receptors for axon guidance cues and play synaptic roles (González-Calvo et al., 2022). Moreover, a CUB domain is engaged in the “Venus-flytrap” mechanism of inhibition of endopeptidases by the human pan-peptidase tetrameric inhibitor α_2_-macroglobulin. It participates in major structural rearrangement of the C-terminal half of the protomer, which further includes three more domains (Marrero et al., 2012; Goulas et al., 2017; Luque et al., 2022). A CUB domain also participates in the “snap-trap” mechanism of monomeric α_2_-macroglobulin-related inhibitors from commensal and pathogenic bacteria such as *Escherichia coli* and *Salmonella enterica* (Wong and Dessen, 2014; Garcia-Ferrer et al., 2015; Goulas et al., 2017).

Next, EGF domains (Pfam-00008) are widely present in up to six copies in several astacins, including meprins, BTPs and proteins from nematodes and echinoderms (Figure 1). Generally, they are found in many animal proteins in the extracellular part of membrane-bound or secreted proteins (Bork et al., 1996). In meprin β, the EGF-like domain is considered a hinge domain, which moves the dimer from a membrane-proximal position for cleavage of transmembrane substrates, such as the amyloid precursor protein, to a membrane-distal position upon binding to its endogenous inhibitor fetuin B (Karmilin et al., 2019; Eckhard et al., 2021). We obtained a generally reliable *AlphaFold* computational model (see the Methods section) for the EGF-like domain of human BMP1 (see Figure 2E). It revealed a ∼40-residue structure cross-connected by three disulfide bonds for structural integrity and two β-hairpins, which overall conform to the standard architecture of these domains (Wouters et al., 2005).

Finally, large diversity is found across astacin PROs, which range between 34 and 486 residues and just share the motif FXGDI among animal orthologues (Guevara et al., 2010; Gomis-Rüth et al., 2012a). The PROs of crayfish astacin (Figure 2F), human meprin β (Figure 2G), bacterial myroilysin (Figure 2H) and horseshoe crab astacin (Figure 2I) have been structurally characterized. They revealed essentially unstructured peptides running along the cleft of the CD in the opposite direction of a true substrate, which precludes their intramolecular cleavage (Guevara et al., 2010; Arolas et al., 2012; Xu et al., 2017; Guevara et al., 2022). The catalytic solvent molecule bound to the catalytic zinc ion in the mature CD (Figure 2A) is replaced by either the aspartate of the motif in the three animal zymogens (Guevara et al., 2010; Arolas et al., 2012; Guevara et al., 2022) or a cysteine in the bacterial enzyme (Xu et al., 2017), which lacks the motif. These residues operate according to an “aspartate-switch” or “cysteine-switch” mechanism of latency, respectively. Such mechanisms have been also reported, among others, for the MPs fragilysin-3 from *Bacteroides fragilis* (Goulas et al., 2011) and matrix metalloproteinases (Springman et al., 1990; Rosenblum et al., 2007), respectively.

### 2.2 Astacins possibly originate in Holozoa

**FIGURE 3:**
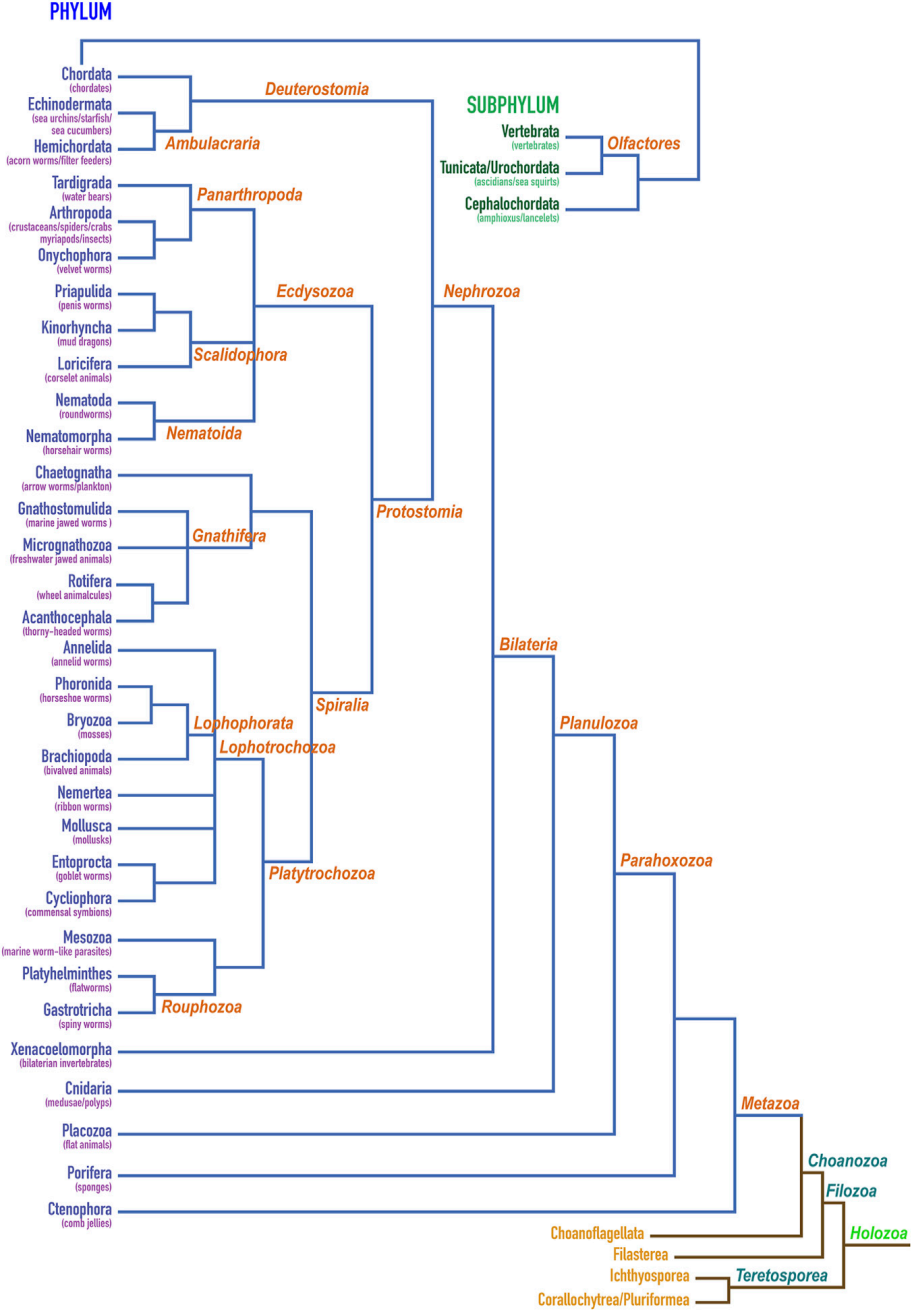
Classification of holozoans. Dendrogram depicting the herein proposed hierarchical clustering of phyla within holozoans assembled based on current literature (Ryan et al., 2010; Ruggiero et al., 2015; Torruella et al., 2015; Cannon et al., 2016; Lu et al., 2017; Sebé-Pedrós et al., 2017; Whelan et al., 2017; Adl et al., 2019; Giribet et al., 2019; Laumer et al., 2019; Marlétaz et al., 2019; Sogabe et al., 2019; Hickman et al., 2020; Schoch et al., 2020; Schulze and Kawauchi, 2021). Phylum Chordata is further shown for its constituting subphyla Vertebrata, Tunicata/Urochordata and Cephalochordata. The first two give rise to Olfactores.

Multicellularity presumably originated several times in unicellular opisthokont holozoans, which have been suggested as precursors of metazoans [(Sebé-Pedrós et al., 2017; Berman 2019)]. To better understand the hierarchical clustering of the distinct phyla within Holozoa, which originate 1.3 billion years ago (Berman 2019), and to put our phylogenetic studies into context, we tentatively assembled a consensus dendrogram based on current literature (Figure 3) given the apparent disparity in the available models (see Section 3.2). This hypothesis entails that Holozoa would split into Teretosporea, themselves consisting of Corallochytrea/Pluriformea (alias Opisthokonta incertae sedis) and Ichthyosporea, and Filozoa. These, in turn, would divide into Filasterea and Choanozoa. The latter would consist of Choanoflagellata, which are unicellular flagellates, and Metazoa, which encompass the multicellular animals and date back to about 760 million years ago (Berman 2019). Up the tree, Bilateria would englobe animals with a plane of symmetry (including Xenacoelomorpha), except echinoderms, which evince post-larval (secondary) pentaradial symmetry. They sequentially would team up with Cnidaria, Placozoa, Porifera and Ctenophora to eventually form Metazoa (Figure 3).

**FIGURE 4:**
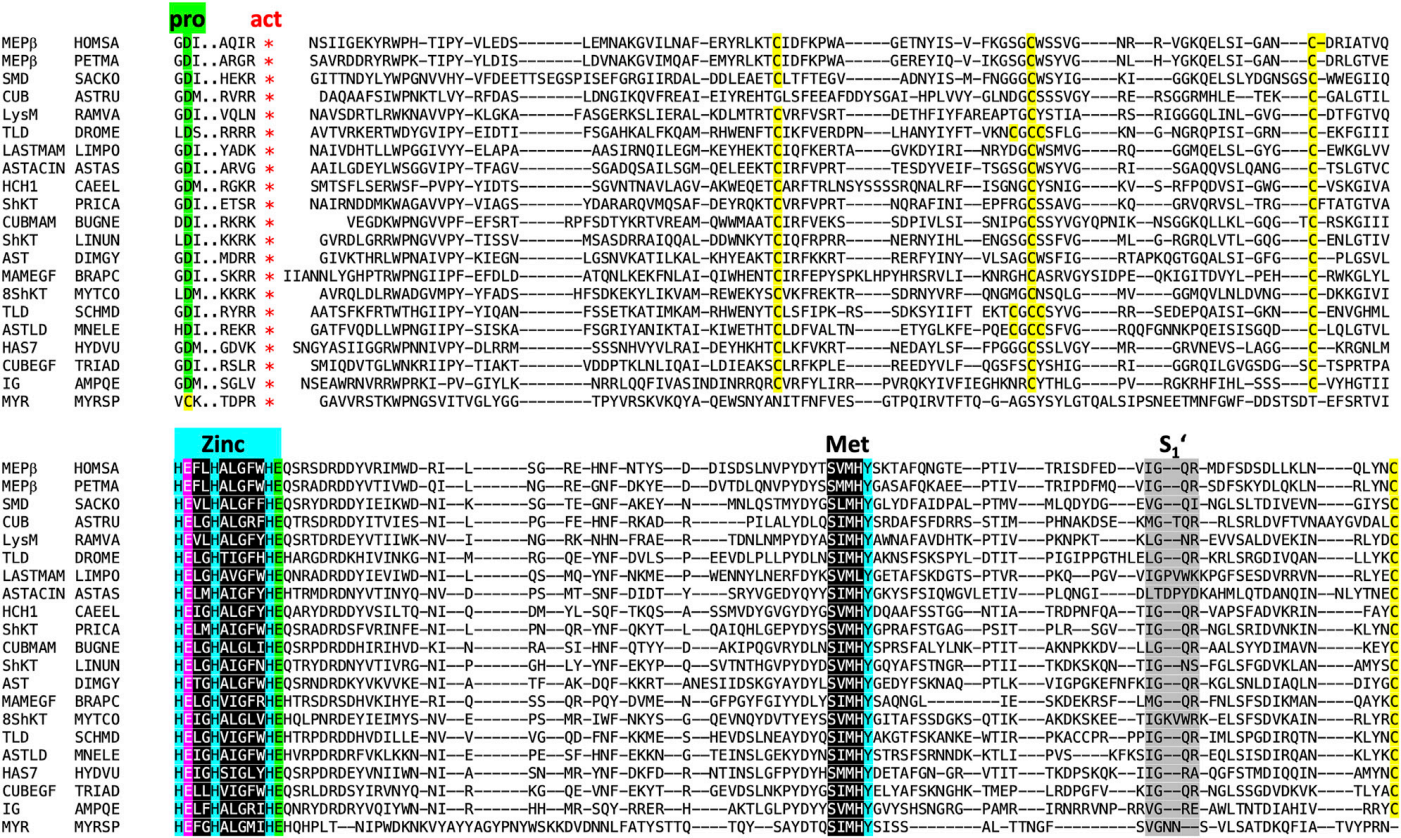
Sequence alignment of 20 astacins representing different animal phyla excerpted from the sets shown in Figure 5, Table 1 and Supplementary Table S1. In the headline, “pro” labels three residues of the prodomain with the conserved aspartate residue (underlaid green) responsible for latency of the proenzyme, which is absent from myroilysin. The red asterisks below “act” indicate the site of proteolytic activation (maturation). Cysteines are underlaid yellow. “Zinc” labels the three zinc-binding histidines (underlaid blue), the catalytically essential glutamate (Arolas et al., 2018) following the first histidine is underlaid magenta, and the family-specific glutamate after the third histidine in underlaid green. Its negatively charged sidechain forms a salt bridge with the positively charged mature amino terminus after activation. Also labelled is the Met-turn (“Met”, underlaid black) with the tyrosine zinc ligand underlaid blue. Finally, S_1_’ indicates the region shaping the binding pocket of the P_1_’ residue of substrates within the catalytic cleft (Schechter and Berger, 1967; Gomis-Rüth et al., 2012b). Sequences: MEPβ HOMSA (*Homo sapiens*, human, phylum Chordata, subphylum Vertebrata), MEPβ PETMA (*Petromyzon marinus*, lamprey, phylum Chordata, subphylum Vertebrata), SMD SACKO (*Sackoglossus kowalevskii*, acorn worm, phylum Hemichordata), CUB ASTRU (*Asterias rubens*, sea star, phylum Echinodermata), LysM RAMVA (*Ramazottius varieornatus*, water bear, phylum Tardigrada), TLD DROME (*Drosophila melanogaster*, fruit fly, phylum Arthropoda, subphylum Hexapoda), LASTMAM LIMPO (*Limulus polyphemus*, horseshoe crab, phylum Arthropoda, subphylum Chelicerata), AST ASTAS (*Astacus astacus*, crayfish, phylum Arthropoda, subphylum Crustacea), HCH1 CAEEL (*Caenorhabditis elegans*, nematode, phylum Nematoda), ShKT PRICA (*Priapulus caudatus*, cactus worm, phylum Priapulida), CUBMAM BUGNE (*Bugula neritina*, common bugula, phylum Bryozoa), ShKT LINUN (*Lingula unguis*, lamp shell, phylum Brachiopoda), AST DIMGY (*Dimorphilius gyrociliatus*, phylum Annelida), MAMEGF BRAPC (*Brachionus plicatilis*, rotifer, phylum Rotifera), ShKT8 MYTCO (*Mytilus coruscus*, Korean mussel, phylum Mollusca), ShKT SCHMD (*Schmidtea mediterranea*, triclad flatworm, phylum Platyhelminthes), ASTLD MNELE (*Mnemiopsis leidyi*, sea walnut, Phylum Ctenophora), HAS7 HYDVU (*Hydra vulgaris*, hydra, phylum Cnidaria), CUBEGF TRIAD, *Trichoplax adherens*, flat-bodied animal, phylum Placozoa and IG4 AMPQE (*Amphimedon queenslandica*, sponge, phylum Porifera). In addition, an astacin xenologue from the bacterium *Myroides* sp., the only known astacin with a proven cysteine switch activation mechanism (Xu et al., 2017; Ran et al., 2020), was further included for comparison (MYR MYRSP).

We performed searches for astacins in several protein and gene databases (see Section 3.1), which revealed > 25,000 entries for potential peptidases of the M12A family. This is how astacins are defined in the MEROPS database of peptidases and their inhibitors [www.ebi.ac.uk/merops; (Rawlings and Bateman, 2021)]. In addition, > 12,000 sequences from > 1,000 species of identified and putative family members were found within family PF01400 within the PFAM database (Mistry et al., 2021). At this point, high-confidence manually curated sequence searches were performed with the sequence of the mature CD of crayfish astacin. The resulting hit sequences were verified to span the entire CD and contain the intact zinc-binding motif, as well as the family-specific glutamate followed by the XXRXDRD and SIMHY motifs, with just minimal conservative substitutions (Figure 4 reproduces selected aligned example sequences). They were further checked to contain a PRO with the zinc-blocking aspartate. A subgroup of sequences was chosen for alignments, phylogenetic tree construction and physiological considerations (Supplementary Table S1; Sections 2.4–2.7). In addition, Table 1 presents a selection of described and potential non-vertebrate metazoan astacins.

**TABLE 1:** Representative sequences of studied and potential non-vertebrate metazoan astacins.

*Phylum Chordata*
*Subphylum Tunicata/Urochordata*
Sea squirt *Halocynthia roretzi* Cho et al. (2008); base tunicate *Ciona intestinalis* Davis and Smith (2002)
*Subphylum Cephalochordata*
Florida lancelet *Branchiostoma floridae* UP C3Y5H0; lancelet/amphioxus *Branchiostoma belcheri* UP A0A6P5AEI0, UP A9JR45, UP A0A6P4Z1P4, UP A0A6P5ALI5
*Phylum Echinodermata*
Sea urchin *Paracentrotus lividus* Lepage et al. (1992); sea urchin *Strongylocentrotus purpuratus* Reynolds et al. (1992); sea cucumber *Holothuria glaberrima* Mashanov et al. (2012); sea star *Asterias rubens* GB OP067654.1
*Phylum Hemichordata*
Acorn worm *Saccoglossus kowalevskii* Freeman et al. (2012)
*Phylum Tardigrada*
Water bear *Ramazzotius varieornatus* UP A0A1D1VZ89, UP A0A1D1VI43; moss piglet *Hypsibius exemplaris * GB OWA53846; water bear *Hypsibius dujardini* UP A0A1W0WK06
*Phylum Arthropoda*
*Subphylum Crustacea*
Crayfish *Astacus astacus* Stöcker et al. (1990); Kamchatka crab *Paralithodes camtschatica* Semenova et al. (2006); common fish louse *Argulus foliaceus* AmbuAli et al. (2020); water fleas *Daphnia pulex* Schwerin et al. (2009) and *Daphnia magna* UP A0A0P6DSE7, UP A0A0P5US34, UP A0A0P6HCB0; shrimp *Penaeus vannamei* UP A0A423TAW0, UP A0A3R7NMP2, UP A0A3R7N8Y5
*Subphylum Chelicerata*
Brazilian brown recluse spider *Loxosceles intermedia* da Silveira et al. (2007); Chilean/Peruvian recluse spider *Loxosceles laeta* (Medina-Santos et al., 2019); yellow garden spider *Argiope aurantia* Foradori et al. (2006); Adanson’s house jumper *Hasarius adansoni* Diab et al. (2021); mygalomorph spider *Actinopus* spp. Prosdocimi et al. (2011); orbweaver spider *Gasteracantha cancriformis* Prosdocimi et al. (2011); Brazilian tarantula *Grammostola iheringi* Borges et al. (2016); spitting spider *Scytodes thoracica* Zobel-Thropp et al. (2014a); brown widow spider *Latrodectus geometricus* Khamtorn et al. (2020); black widow spider *Latrodectus hesperus* UP E7D164; cellar spider *Physocyclus mexicanus* Zobel-Thropp et al. (2019); wolf spider *Pardosa pseudoannulata* Huang et al. (2018); Brazilian white-knee tarantula *Acanthoscurria geniculata* and African social velvet spider *Stegodyphus mimosarum* Walter et al. (2017); African hermit spider *Nephilengys cruentata* Fuzita et al. (2015a); primitive hunting spider *Plectreurys tristis* Zobel-Thropp et al. (2014b); yellow scorpion *Tityus serrulatus* Fuzita et al. (2015b); horseshoe crab *Limulus polyphemus* (Becker-Pauly et al., 2009); wheat curl mite *Aceria tosicella* UP A0A8B8R4B3
*Subphylum Hexapoda*
Fruitfly *Drosophila melanogaster* Shimell et al. (1991); sand fly *Lutzomyia longipalpis* Jochim et al. (2008); maybeetle *Melolontha melolontha* Wagner et al. (2002); tse-tse fly *Glossina morsitans* Yan et al. (2002); fall armyworm *Spodoptera frugiperda* Ferreira et al. (2007); Bertha armyworm *Mamestra configurata* Toprak et al. (2016); silkworm *Bombyx mori* Lu et al. (2010); Chinese oak silkworm *Antheraea pernyi* (Tang et al. (2011a); Chinese wild silkworm *Bombyx mandarina* Tang et al. (2011b); Australian sheep blowfly *Lucilia cuprina* Young et al. (2000); ixodid cattle tick *Rhipicephalus microplus* Barnard et al. (2012); American dog tick *Dermacentor variabilis* Sonenshine et al. (2011); spinybacked orbweaver *Gasteracantha cancriformis* (Prosdocimi et al. (2011); African malaria mosquito *Anopheles gambiae* Riehle et al. (2002); yellow-fever mosquito *Aedes aegypti* UP Q17KW5, UP Q16JR6; sandfly *Phlebotomus kandelakii* A0A6B2EJJ9; biting mite *Culicoides sonorensis* UP A0A336L7R2
*Phylum Priapulida*
Cactus worm *Priapulus caudatus* GB XP_014678976
*Phylum Nematoda*
Nematode *Caenorhabditis elegans* Möhrlen et al. (2003), Park et al. (2010); parasite nematode *Trichinella spiralis* Lun et al. (2003); roundworm *Onchocerca volvulus* Borchert et al. (2007); dog hookworms *Ancylostoma caninum* Williamson et al. (2006) and *Ancylostoma ceylanicum* Baska et al. (2013); brown stomach worm *Teladorsagia circumcinta* Stepek et al. (2015); New World hookworm *Necator americanus* Ranjit et al. (2006); brown stomach worm *Ostertagia ostertagi* de Maere et al. (2005); rat small-intestine nematode *Strongyloides ratti* Soblik et al. (2011); human small-intestine nematode *Strongyloides stercoralis* Gómez Gallego et al. (2005), Varatharajalu et al. (2011); interstine nematode *Strongyloides venezuelensis* Yoshida et al. (2011); threadworm *Strongyloides papillosus* Hunt et al. (2016); possum roundworm *Parastrongyloides trichosuri* Hunt et al. (2016); free-living nematode *Rhabditophanes* sp. Hunt et al. (2016); rat hookworm *Nippostrongylus brasiliensis* Sotillo et al. (2014); entomoparasitic nematode *Steinernema carpocapsae* Jing et al. (2010); barber’s pole worm *Haemonchus contortus* Stepek et al. (2010); Stepek et al. (2011); roundworm *Brugia malayi* Stepek et al. (2010); Stepek et al. (2011); roundworm *Angiostrongylys cantonensis*; roundworms *Pristionchus pacificus*, *Meloidogyne hapla*, and *Meloidogyne incognita* Park et al. (2010); rodent roundworm *Heligmosomoides polygyrus* Hewitson et al. (2011). See also Martín-Galiano and Sotillo (2022).
*Phylum Rotifera*
(Bdelloid) rotifers *Brachionus plicatilis* GB RNA28629, GB RNA07860, GB RNA15157; *Brachionus ibericus* GB ADR79275; and *Brachionus calyciflorus* GB CAF0958500, GB CAF0895916, GB CAF0832385; *Adineta ricciae* GB CAF0946005, GB CAF0977041, GB CAF0768045; *Adineta vaga* GB UJR10667, GB UJR30378, GB UJR12793; *Adineta steineri* GB CAF1145707, GB CAF0769233, GB CAF1137664; *Rotaria* sp. Silwood-1/2 GB CAF3391332, GB CAF2377320, GB CAF4963388; *Rotaria sordida* GB CAF0972930, GB CAF1460355, GB CAF1002947; *Rotaria magnacalcarata* GB CAF1378535, GB CAF4188303, GB CAF5207147; *Rotaria socialis* GB CAF3283866, GB CAF3682864, GB CAF4691708; *Didymodactylos carnosus* GB CAF1098425, GB CAF0882139, GB CAF1230341
*Phylum Annelida*
Tubeworm *Owenia fusiformis* GB CAC9583006, GB CAH1790161, GB CAH1785993; meiofaunal worm *Dimorphilus gyrociliatus* GB CAD5123995, GB CAD5123793, GB CAD5125857; leech *Helobdella robusta* GB XP_009017824, GB XP_009026841, GB XP_009025904; segmented worm *Capitella teleta* GB ELT87968, GB ELU00391, GB ELU00287; Satsuma tubeworm *Lamellibrachia satsuma* GB KAI0241173, GB KAI0224812, GB KAI0229203
*Phylum Bryozoa*
Common bugula *Bugula neritina* GB KAF6036083, GB KAF6021779, GB KAF6027899
*Phylum Brachiopoda*
Common oriental lamp shell *Lingula anatina* GB XP_013381380, GB XP_013399408, GB XP_013417809; lamp shell *Lingula unguis* UP A0A1S3HHT9, UP A0A1S3KFN6, UP A0A1S3IEM6
*Phylum Mollusca*
Sea hare *Aplysia californica* Liu et al. (1997); spear squid *Loligo bleekeri* Yokozawa et al. (2002); Akoya pearl oyster *Pinctada fucata* Xiong et al. (2006); Pacific oyster *Crassostreas gigas* (Roberts et al., 2009); Suminoe oyster *Crassostrea ariakensis* (Yang and Wu, (2009); myosinase from the Japanese flying squid *Todarodes pacificus* Yokozawa et al. (2002); vampire snail *Cumia reticulata* Gerdol et al. (2019); golden cuttlefish *Sepia esculenta* Kumar et al. (2018); bigfin reef squid *Sepioteuthis lessoniana* Kanzawa et al. (2008); spear squid *Loligo bleekeri* Kanzawa et al. (2005); Mediterranean mussel *Mytilus galloprovincialis* Yu et al. (2016); *Mytilus coruscus*, Korean mussel UP A0A6J8APQ9, UP A0A6J8DI28, UP A0A6J8BR94, UP A0A6J8CIB2, UP A0A6J7ZT88, UP A0A060Q6V2
*Phylum Platyhelminthes*
Triclad flatworms *Schmidtea mediterranea* Isolani et al. (2013), UP A0A4Z2DK01 and *Dugesia japonica* Isolani et al. (2013); blood flukes *Schistosoma mansoni* and *Schistosoma japonicum* Park et al. (2010); liver fluke *Fasciola hepatica* UP A0A4E0RFD2; tapeworm *Echinococcus multilocularis* UP A0A068Y1M8. See also Martín-Galiano and Sotillo (2022).
*Phylum Xenacoelomorpha*
Parasitic aquatic worm *Meara stichopi* GB AVK72361; panter worm *Hofstenia miamia* GB AID23683
*Phylum Cnidaria*
Fresh-water polyp *Hydra vulgaris* Sarras et al. (2002); hydroid *Hydractinia echinata* Möhrlen et al. (2006); jellyfish *Podocoryna carnea* Pan et al. (1998); carp endoparasite *Sphaerospora molnari* Hartigan et al. (2020); blade fire coral *Millepora complanata* Hernández-Elizárraga et al. (2019); lion’s mane jellyfish *Cyanea capillata* Liu et al. (2015); Nomura’s jellyfish *Nemopilema nomurai* Kang et al. (2014); sea anemone *Nematostella vectensis* Möhrlen et al. (2006)
*Phylum Placozoa*
Flat-bodied animal *Trichoplax adhaerens* GB XP_002110676, GB XP_002110677, GB XP_002113592 and *Trichoplax* sp. H2 GB RDD40966, GB RDD38391, GB RDD44281
*Phylum Porifera*
Sponge *Amphimedon queenslandica* GB XP_019850969, GB XP_019859271, GB XP_019850968
*Phylum Ctenophora*
Sea walnut *Mnemiopsis leidy* GB AEP16401
*Bacterial astacins* ^a^
*Flavobacterium meningosepticum* flavastacin [UP Q47899; Tarentino et al. (1995)]; myroilysin from *Myroides profundi * (UP B5B0E6) and *Myroides* sp. CSLB8 (UP A0A0P0DZ84) Xu et al. (2017), Ran et al. (2020)

GB, *GenBank* code (www.ncbi.nlm.nih.gov/genbank); UP, *UniProt* code (www.uniprot.org). Phyla are consistent with Figure 3.

**FIGURE 5:**
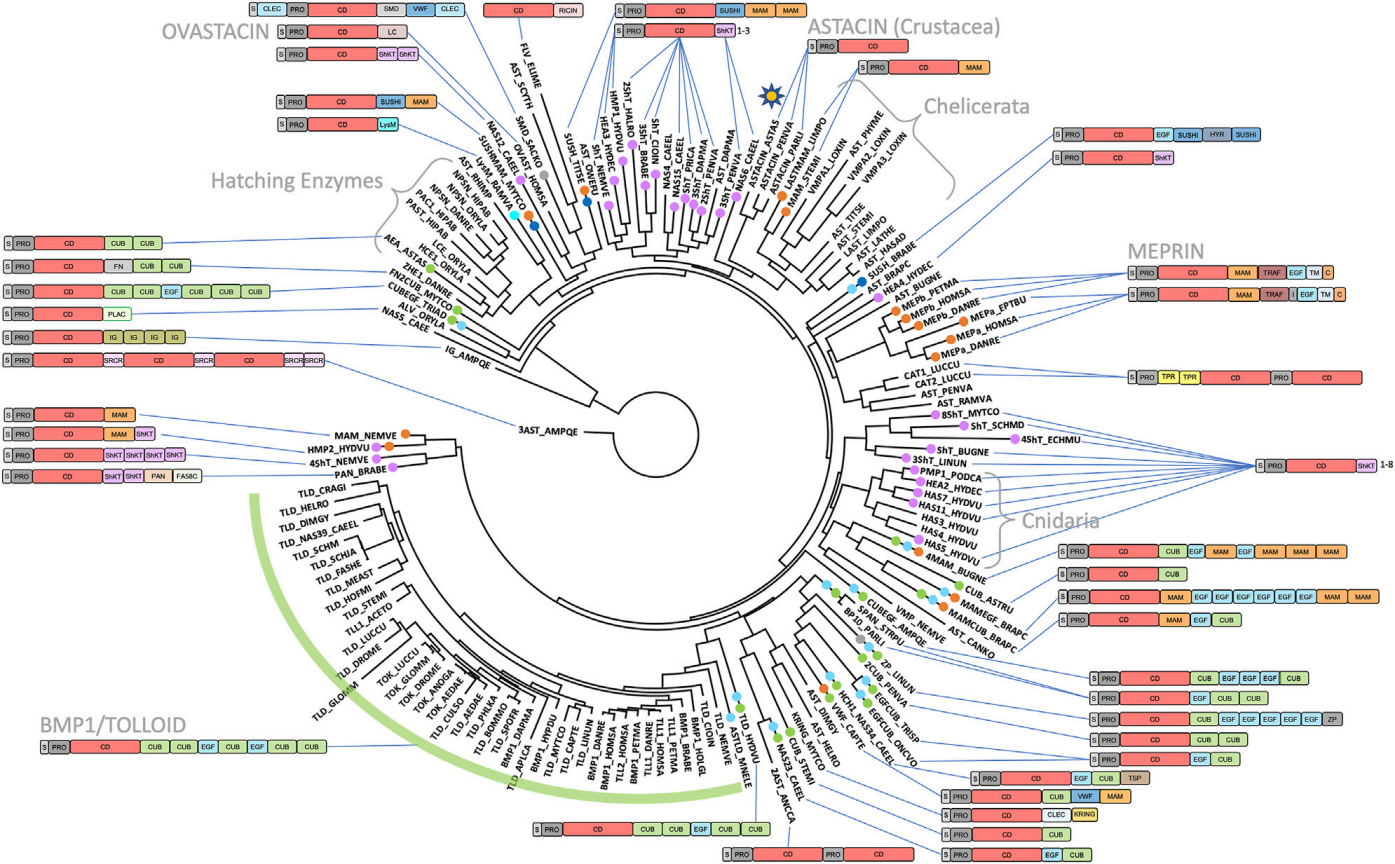
Phylogenetic tree based on the catalytic domains of a selection of 147 astacins. The list of species and *UniProt* and *GenBank* accession numbers are listed in Supplementary Table S1. The asterisk in the top right quadrant indicates the position of the prototypical name-giving enzyme astacin from the crayfish *Astacus astacus*. Crayfish astacin is translated with a signal peptide for extracellular targeting, a prodomain conferring latency and a catalytic protease domain (see also Figure 1). The domain compositions of astacins consisting merely of these three domains are omitted for clarity. Astacin-like proteases with more complex domain structures are shown schematically. A detailed list of domains with *Prosite* database accession numbers is contained in Figure 1 and in Supplementary Table S1.

^a^ Despite not being from metazoans, these proteins have been listed as they are the only biochemically and structurally characterized astacins outside Holozoa. They appear in Figures 1, 5.

We consistently found astacin sequences from humans down the tree of life until the root of subphylum Vertebrata (a selection is provided by Supplementary Table S1), among which the jawless fishes (Agnatha) are most basal, with two extant genera: lampreys and hagfishes (Supplementary Table S1). Vertebrata associate with the subphyla Tunicata/Urochordata, which includes sea squirts and the base tunicate *Ciona intestinalis*, and Cephalochordata, which features the lancelet (amphioxus), to form the phylum Chordata (Figure 3). These taxa also evinced abundant astacins. In addition, we could find sequences for the phyla Echinodermata and Hemichordata within Ambulacraria, which together with Chordata form Deuterostomia. Within Ecdysozoa, we could retrieve sequences from Tardigrada and Arthropoda but not Onychophora within Panarthropoda; Priapulida but not Kinorhyncha or Loricifera within Scalidophora; and Nematoda but not Nematomorpha within Nematoida. Out of Gnatifera plus Chaetognatha, we could only get hits for Rotifera. As to Lophotrochozoa, we found astacins in Annelida, Bryozoa, Brachiopoda and Mollusca. Next, we identified potential orthologues within Platyhelminthes but not Mesozoa and Gastrotricha. All these phyla constitute Protostomia, which together with Deuterostomia form Nephrozoa (Figure 3). The latter give rise to Bilateria together with Xenacoelomorpha, for which we could find sequences in two organisms (Table 1). Finally, we could identify astacins in all the remaining more primitive metazoan phyla: Cnidaria, Placozoa, Porifera and Ctenomorpha. All these results (Figure 3; Table 1) suggest that astacins are systematically present at least down to the root of Metazoa.

Choanoflagellata have been discussed as animal precursors for a long time, since they are similar to choanocytes of sponges (Hickman et al., 2020). However, a search for astacins within *Monosiga brevicolis* (King et al., 2008), *Salpingoeca rosetta* [formerly *Proterospongia* sp.; (Fairclough et al., 2013)] and *Monosiga ovata* of this phylum did not reveal significantly similar proteins. In addition, organisms from the next related taxa, Filasterea (*Capsaspora owczarzaki*), Ichthyosporea (*Sphaeroforma arctica*) and Corallochytrea/Pluriformea (*Corallochytrium limacisporum* and *Syssomonas multiformis*), which together with Metazoa and Choanoflagellata constitute the Holozoa (Figure 3), did not contain potential orthologs either in the generally available databases. At this point, we got access to unpublished genome sequences from a series of organisms at the root of Holozoa, viz., the Choanoflagellata *Acanthoeca spectabilis, Choanoeca perplexa, Diaphanoeca grandis, Salpingoeca dolichothecata, Salpingoeca infusorum, Salpingoeca kvevrii, Salpingoeca macrocollata, Salpingoeca punica, Salpingoeca urceolata* and *Stephanoeca diplocostata*; the Filastereum *Ministeria vibrans*; and the Ichthyosporea *Amoebidium parasiticum, Abeoforma whisleri* and *Pirum gemmata* [I. Ruiz-Trillo & M. Leger, personal communication, and (Torruella et al., 2015; Carr et al., 2017; Grau-Bové et al., 2017)]. We identified several potential astacin orthologues in *Diaphanoeca grandis* (e.g., comp29462_c0_seq1:1552770, comp29716_c0_seq2:1462332 and comp33115_c0_seq4:851467), *Salpingoeca dolichothecata* (e.g., comp24492_c1_seq1:7011852, comp14956_c0_seq2:1981400 and comp26781_c0_seq6:13261), *Amoebidium parasiticum* (Amoebidium_parasiticum_Apar_comp21710_c1_seq1m30654/1239) and *Ministeria vibrans* (Ministeria_vibrans_Mvib_g2222t1/16202), whose sequences are provided in Supplementary Figure S1. Altogether, these findings would suggest that astacins probably originate in the root of Holozoa according to Darwinian descent.

A selection of invertebrate and vertebrate sequences (Supplementary Table S1) enabled us to construct a phylogenetic tree for metazoan astacins (Figure 5), which was solely based on a sequence alignment of the respective CDs. Three sequences encompassed multiple CDs, which originate in the most basal sponge *Amphimedon qeeenslandica* (*UniProt* code [UP] A0A1X7U9V1), the nematode hookworm *Ancylostoma caninum* (UP A0A368GTC8) and the blow fly *Lucilia cuprina* (UP A0A0L0BYD0). This tree does not mirror the phylogenesis of organisms presented in Figure 3 since it contains both orthologous and paralogous astacins. Indeed, members of the distinct subfamilies evinced separate clustering (Figure 5). The tree is not comprehensive, since only a selection of astacins comprising the minimal setup of typical sequential and structural motifs (as outlined above) were included. Nevertheless, certain clusters of astacins can be recognized. These are, starting clockwise in the upper left quadrant of the circular tree (Figure 5), the 1) hatching enzymes, which originate from the same root as ovastacin; 2) ShKT-carrying astacins from chordates, nematodes, cnidarians, priapulids and arthropods (chelicerates and crustaceans), which include the prototypal crayfish astacin; 3) a clade containing the meprins; 4) a second cluster of ShKT-astacins, mostly from cnidarians (right centre); 5) proteins rich in MAM, EGF and CUB domains (right bottom); and 6) the BTPs (left bottom).

### 2.3 Scattered presence of astacins outside Holozoa

Searches in non-holozoan Eukaryota including plants, fungi and the fungus-like Oomycota revealed merely < 400 sequences scattered across Alveolata, Stramenopila, Rhizaria, Archaeplastida (Haptista), Excavata (Discoba) and some Amoebozoa clades. By contrast, no astacins were detected in any of the other eukaryotic taxa except for the silver mallet wood *Rhodamnia argentea* [*GenBank* code (GB) XP_030553468], the only plant orthologue retrieved. However, this entry was debunked as a contamination with a tolloid-like chelicerate astacin from the wheat curl mite *Aceria tosichella* (UP A0A8B8R4B3).

The observation of astacin-like proteins within Stramenopila is remarkable since this taxon alone already accounts for ∼200 of the hits. These are heterokonts that were formerly grouped into fungi (Holomycota) but currently are considered to be closer to brown algae than to fungi (Sebé-Pedrós et al., 2017). They further include the Oomycetes (“egg fungi”), a clade containing many parasitic/saprophytic organisms, which in turn encompass most of the hits within Stramenopila. Among them is *Aphanomyces astaci*, a well-known parasite of the North American crayfish *Cambarus clarkii*, which developed resistance against this pest. However, when American crayfish were brought to Europe in the late 19th century, *A. astaci* infection caused the “crayfish plague” in the endogenous crayfish population (*A. astacus*), which almost caused its extinction (Diéguez-Uribeondo and Söderhäll, 1993). Oomycetes are known champions of horizontal gene transfer (HGT) (Koonin et al., 2001; Keeling and Palmer, 2008), thereby gathering enzymes useful to target their prey (Judelson, 2017). This genetic transfer route could thus explain the generally scattered but locally focused presence of astacin CDs, which is inconsistent with Darwinian descent, in eukaryotes outside Holozoa.

HGT could also account for the sporadic occurrence of astacin-like peptidases in archaea, bacteria and viruses, which would thus also be xenologues (Koonin et al., 2001). Examples of archaeal sequences were found in *Candidatus korarcheota* (UP A0A662SFB1), *Nitrosopumilus* sp. (GB MCA9827382), *Methanotrichaceae archaeon* (GB MBN1323470) and *Halobacteriales archaeon* QH_6_64_20 (GB PSP40402). Viral sequences were restricted to Lutzomyia reovirus 2 (UP A0A0H4M9A8). The more populous bacterial examples were from *Bacillus cereus* (GB WP_235610182), *Acinetobacter baumanii* (GB WP_207273295), *Klebsiella pneumoniae* (GB NAU77905), *Bacillus thuringiensis* (GB WP_228528809), *Legionella pneumophila* (GB WP_061484376), *Bacillus mycoides* (GB WP_186320991), among others. Overall, the vast majority of bacterial astacin hosts live in intimate contact with animals, which would facilitate HGT of genes from eukaryotes to prokaryotes. Among them are those of the biochemically studied proteins flavastacin from *Flavobacterium meningosepticum* [also known as *Elizabethkingia meningoseptica*; UP Q47899; (Tarentino et al., 1995)] and myroilysins from *Myroides profundi* (UP B5B0E6) and *Myroides* sp. CSLB8 (UP A0A0P0DZ84) (Xu et al., 2017; Ran et al., 2020). In the latter case, zymogenic latency was shown to follow a different mechanism from the animal forms [see Section 2.1; (Guevara et al., 2022)], which would further support an HGT event as the origin of its presence in the bacterium. This is reminiscent of the aforementioned fragilysin-3, which originates in a member of the human colon microbiota. Its CD was proposed to be an adamalysin/ADAM xenologue acquired by HGT from the host that separately evolved to derive a distinct mechanism of latency (Goulas et al., 2011; Goulas et al., 2013).

### 2.4 Functional and evolutionary aspects of BMP1/tolloid-like peptidases

In the phylum Chordata, subphylum Vertebrata, order Mammalia, six genes encode astacin peptidases [*bmp1, tll1, tll2, mepa, mepb* and *astl*; http://degradome.uniovi.es/met.html; (Quesada et al., 2009)]. The first three genes code for the BMP1, TLL1 and TLL2 proteins, which belong to the BTPs (Wozney et al., 1988; Nguyen et al., 1994). They are characterized by an arrangement of five CUB domains and two EGF-like domains C-terminal of the CD and are present in virtually all metazoan phyla (Figures 1, 5). BTPs are important for dorsoventral patterning in metazoan embryogenesis (Sato and Sargent, 1990; Holley et al., 1996; de Robertis, 2008; de Robertis and Tejeda-Muñoz, 2022). In deuterostomes, they are also crucial for extracellular matrix assembly through the processing of precursors of matrix components, growth factors and their receptors (Kessler et al., 1996; Ge and Greenspan, 2006). The CUB and EGF-like domains of these astacins bear important exosites for substrate recognition and targeting toward the extracellular matrix (Sieron et al., 2000; Garrigue-Antar et al., 2004; Ge and Greenspan, 2006; Hintze et al., 2006; Hopkins et al., 2007; Wermter et al., 2007). Although BTPs are closely linked with bilaterian morphogenesis, which supposedly originated in “Urbilateria” (de Robertis, 2008; de Robertis and Tejeda-Muñoz, 2022), they are not restricted to Bilateria and are also present in radially symmetric metaozans, Cnidaria (Saina et al., 2009) and Ctenophora (Pang et al., 2011). Related proteins with slightly deviating CUB/EGF arrangements were also found in the basal placozoan species *Trichoplax adhaerens* (CUBEGF_TRIAD) and in several other astacins (Figure 5). Taken together, BTPs are pan-metazoan astacins with essential functions in embryogenesis, which exert further additional functions in several metazoan phyla.

### 2.5 Functional and evolutionary aspects of meprin metallopeptidases

Two other human astacin genes, *mepa* and *mepb*, encode meprin α and meprin β for which orthologs have only been detected among vertebrates. Both meprins are membrane bound but meprin α is released already in the trans-Golgi network by furin cleavage and stays membrane bound only in association with meprin *β*. The latter is a “sheddase”, which releases cell-surface proteins such as growth factors, cytokines, receptors, as well as amyloid precursor protein through cleavage at its β-secretase site. Deregulation of meprins leads to neurodegenerative diseases, changes in barrier function (such as in the blood brain barrier), inflammatory bowel disease, fibrosis, nephritis and cancer (Sterchi et al., 2008; Arolas et al., 2012; Becker-Pauly and Pietrzik, 2016; Arnold et al., 2017; Eckhard et al., 2021; Gindorf et al., 2021; Bayly-Jones et al., 2022; Werny et al., 2022). The unique domain composition of meprins includes MAM, TRAF and EGF-like domains (Figures 1, 5), and although MAM- and EGF-containing astacins have been identified in other metazoan phyla (see Figures 1, 5; Supplementary Table S1), none of these are apparently membrane bound or exhibit comparable physiological potential to vertebrate meprins. Finally, our database searches unravelled meprin-like astacin-CDs also in basal vertebrates such as lamprey (MEPβ_PETMA) and hagfish (MEPα_EPTBU), which just encompass the S, PRO, CD and MAM moieties (see Figures 1, 5; Supplementary Table S1), similarly to a reported horseshoe crab enzyme [LASTMAM_LIMPO; (Becker-Pauly et al., 2009; Guevara et al., 2022)].

### 2.6 Astacins in animal reproduction

The sixth human astacin is ovastacin, which is encoded by the *astl* gene and is expressed only in oocytes among mammals (Burkart et al., 2012). Absence of ovastacin results in subfertility, since it is released during the cortical reaction after intrusion of a sperm cell and causes hardening of the zona pellucida of the extracellular matrix surrounding the egg. This provides rigidity and robustness to the resulting embryo until its implantation in the uterus (Stöcker et al., 2014; Körschgen et al., 2017). Ovastacin has the basic domain composition S-PRO-CD, which is followed by a disordered domain of unknown function. This domain stays connected with the oolemma after the release of the enzyme into the perivitelline space during the cortical reaction, which suggests a function in membrane anchoring and shedding of ovastacin (Körschgen et al., 2017). A similar function to ovastacin was reported for alveolin from the medaka fish *Oryzias latipes*, which likewise hardens the envelope of the fertilized egg (zygote) in bony fishes (Shibata et al., 2000).

In egg-laying vertebrates like fishes, amphibians, reptiles and birds, a specialized group of astacin MPs termed hatching enzymes has evolved (Nagasawa et al., 2022). They are absent from mammals and involved in the cleavage of the eggshell. They optionally contain an additional pair of cysteine residues in the N-terminal subdomain of their mature CDs compared to crayfish astacin, as well as extra C-terminal CUB domains (Figures 1, 5). Hatching enzymes are also present in egg-laying invertebrates, such as the crayfish *A. astacus*, which in addition to the prototypic digestive astacin archetype possesses the “*Astacus* embryonic astacin” (AEA_ASTAS in Figure 5; Supplementary Table S1; Geier and Zwilling, 1998). Finally, a reproductive astacin was also described from *Drosophila* seminal plasma (SEMP1_DROME; Figure 5; Supplementary Table S1). This MP is involved in a proteolytic cascade that triggers sperm capacitation and thus regulates fertility in the fruitfly (LaFlamme et al., 2012; LaFlamme and Wolfner, 2013; LaFlamme et al., 2014).

### 2.7 Astacins of helminths and cnidarians

The genome analysis of the roundworm *Caenorhabditis elegans* uncovered 40 astacins termed “nematode astacins” (Möhrlen et al., 2003; Park et al., 2010). Moreover, in a comprehensive analysis of 154 helminth species of the phyla Nematoda and Platyhelminthes, many of them parasitic, an enormous radiation of astacins was also observed (Martín-Galiano and Sotillo, 2022). Most remarkable are the > 100 different additional domains that occur downstream of the CD in variable combinations, thus yielding an enormous functional versatility for these proteins. These domains do not only include protein-protein and protein-carbohydrate interacting domains, but also additional enzymatic functions, such as trypsin-like serine peptidases and hydroxylases.

Particularly striking are astacins containing “ShKT” domains, which mimic a toxin from the sea anemone *Stychodactyla helianthus* that blocks potassium channels (Castañeda et al., 1995). We found such ShKT-astacins in Nematoda (CAEEL, ONCVO, ANCCA and TRISP; see Figure 5; Table 1; Supplementary Table S1), Plathyhelminthes (SCHMD, ECHMU and SCHJA), Arthropoda (PENVA and DAPMA), Priapulida (PRICA), Bryozoa (BUGNE and LINUN), Mollusca (MYTCO) and Cnidaria (HYDVU, HYDEC, PODCA, NEMVE), as well as in the lower chordates sea squirt (HALRO and CIOIN) and lancelet (BRABE). In Figure 5, ShKT-astacins are labelled with pink branch tips (see Supplementary Table S1). These astacins are mostly expressed in epithelia forming barriers to the environment or in the digestive tract, which suggests functions in protection and defence, as well as preservation of the epithelial integrity (Park et al., 2010; Isolani et al., 2018). However, considering the parasitic lifestyle of many of the organisms harbouring ShKT-astacins, the combination of proteolytic and toxin domains may also challenge the respective host (Moran et al., 2013; Martín-Galiano and Sotillo, 2022). Similarly, toxicity has also been reported for astacins lacking ShKT domains from spider venoms (Trevisan-Silva et al., 2010), in which other proteins may take over the role of the latter domains in linking proteolytic activity with specific toxicity. This is reminiscent of the snake venom MPs from the adamalysin/ADAM family, for which forms spanning only the CD are not haemorrhagic while those encompassing further C-terminal disintegrin-like and cysteine-rich domains may be haemorrhagic (Fox and Serrano, 2009; Gomis-Rüth et al., 2013; Herrera et al., 2018). Finally, many ShKT-astacins carry additional CUB, EGF, MAM, etc. domains that may serve to modulate activity. Examples are the morphogenetically active *Hydra vulgaris* ShKT-proteins HMP1, HMP2 and HAS7 (Figure 5; Supplementary Table S1). While HMP1 is involved in head formation and head regeneration (Yan et al., 1995), HMP2 has a function in foot morphogenesis (Yan et al., 2000) and HAS7 specifically cleaves *Hydra* WNT3 during head morphogenesis and thereby restricts head organizer formation (Ziegler et al., 2021; Holstein, 2022).

### 2.8 New functions of astacins

A surprising function for an astacin peptidase was very recently reported for the sea star *Asterias rubens*, which underpins the enormous versatility of nature’s toolbox*.* In zoology textbooks [e.g., (Hickman et al., 2020)], the locomotion of Asteroidea on a surface is usually explained as the concerted action of a multitude of tiny sucking pods in the podia of the tube feet lining the oral face of the animal’s arms. However, pod attachment is apparently not based on suction but on a secreted glue consisting of adhesive matrix proteins that are left on the surface after detachment. The latter is mediated by an astacin MP spanning a CD and a CUB domain (CUB_ASTRU; see Figure 5; Supplementary Table S1), which is specifically secreted by de-adhesive gland cells and releases the adhesive material from the surface of the tube feet (Algrain et al., 2022).

## 3 Materials and methods

### 3.1 Database searches

Sequence searches were performed with the *Psi-Blast* program (Altschul and Koonin, 1998) within *UniProt* (https://www.uniprot.org) and *GenBank* (https://www.ncbi.nlm.nih.gov/genbank) using standard parameters, as well as with the *Ensembl* genome browser (http://www.ensembl.org), within the *Choanobase* database (King, 2005) and the *Merops* database [https://www.ebi.ac.uk/merops; (Rawlings and Bateman, 2021)]. Literature searches for described astacins were performed with the keyword “astacin*” within *PubMed* (https://pubmed.ncbi.nlm.nih.gov), which retrieved 342 papers. Hits were manually curated.

### 3.2 Compilation of a dendrogram for holozoans

The currently available trees for holozoans in zoology textbooks such as (Hickman et al., 2020) present several discrepancies with models proposed by recent research publications (Cannon et al., 2016; Giribet et al., 2019; Laumer et al., 2019). These are based on massive sequence data generated in the last decade by increasingly affordable sequencing methods, first through the Illumina MiSeq platform and more recently through MinION sequencing (Santos et al., 2020), which are also partially applicable to natural history collections (Folk et al., 2021). As an example, phylum Chaetognatha was considered a separate clade at the same level as Spiralia and Ecdysozoa, while it is currently envisaged as a sister clade of Gnathifera within Spiralia (Kocot et al., 2017). Also setting phylum Porifera at the root of Metazoa contradicts recent models, which choose Ctenophora for this place (Giribet et al., 2019; Laumer et al., 2019). Finally, recent work proposed new models at the base of Holozoa, with Ichtyosporea and Corallochytrea/Pluriformea forming Teretosporea, which together with Filozoa give rise to Holozoa (Torruella et al., 2015; Arroyo et al., 2020). Accordingly, we assembled a dendrogram for holozoans based on consensus information extracted from these and other recent publications (Ryan et al., 2010; Ruggiero et al., 2015; Torruella et al., 2015; Cannon et al., 2016; Kocot et al., 2017; Lu et al., 2017; Sebé-Pedrós et al., 2017; Whelan et al., 2017; Adl et al., 2019; Giribet et al., 2019; Laumer et al., 2019; Marlétaz et al., 2019; Sogabe et al., 2019; Schoch et al., 2020; Schulze and Kawauchi, 2021).

### 3.3 Computation of alignments and phylogenetic trees

Amino-acid sequence alignments and phylogenetic trees were computed using the *Seaview* program (http://doua.prabi.fr/software/seaview) (Galtier et al., 1996; Gouy et al., 2010). Alignments were performed with *Clustal Omega* within *Seaview* (Sievers et al., 2011) using default parameters. Manual adjustment of the S_1_’ regions in Figure 4 was performed based on overlays of the X-ray crystal structures of crayfish astacin [Protein Data Bank (PDB) access codes 1AST, 1QJI] and zebrafish hatching enzyme 1 (PDB 3LQB). Phylogenetic trees were calculated with *PhyML* using the maximum likelihood approach as implemented in *Seaview* (Guindon et al., 2010). The BLOSUM62 scoring matrix was used and 100 bootstrap replications were computed in each case. Trees were represented with *Figtree* (http://tree.bio.ed.ac.uk).

### 3.4 Computation of three-dimensional structural models

Precise and accurate computational models of specific astacin domains were obtained with the *AlphaFold* program (Jumper et al., 2021; Tunyasuvunakool et al., 2021). To this aim, the program was locally installed in a high-performance computing cluster operated with linux and the amino acid sequences were fed into the program, which was run employing standard parameters. Quality of the predicted models was monitored through the average predicted local distance difference test (pLDD1) value. Values exceeding 90% are considered to originate in high-accuracy models, while those above 80% correspond to generally correct models for the backbone (Tunyasuvunakool et al., 2021). The unique EGF-like domain of human BMP1 (residues 547–590, see UP P13497) was predicted as a generally correct model for the backbone given an average pLDD1 value of 82.5%. Moreover, the first CUB domain of human BMP1 (residues 321–434, see UP P13497) was predicted highly accurately (average pLDD1 = 91.5%). Three-dimensional structure figures were prepared using *Chimera* (Goddard et al., 2018).

## Data Availability

The original contributions presented in the study are included in the article/Supplementary Material, further inquiries can be directed to the corresponding authors upon reasonable request.
