# Supplementary material for: Structural and evolutionary insights into astacin metallopeptidases
Source: Front Mol Biosci. 2023 Jan 4;9:1080836. doi: 10.3389/fmolb.2022.1080836 (PMC9848320; doi:10.3389/fmolb.2022.1080836)
Supplement: Supplementary file 2 [file DataSheet1.DOCX]

***Suppl. Fig. 1 –*** **List of potential non-metazoan astacins within Holozoa.**

>Diaphanoeca_grandis_m.130167/1871 g.130167 ORF g.130167 m.130167 type:complete len:872 () comp29462_c0_seq1:1552770()

MSVPAMAMAMAGAFIITMSSLSAQVQARPIDDFNPDIIGEGFDMLYSLEQYKTLNPDTLVPFEGSCVRLASVDDESVFTLEHVHDIDTCLTQCINFETETQQEMGACEVVVHSDSEIECRILPVTIVGEDVHVEATADRTCYVLAPGRRRTRRDSHASSTSELKSKRAMVPDANTWPDNEIPYVIMTSSSPYSWHANGVDQTQALQYLNQAFGVYHTLTNIKFRPRDGDAHYIKIGYFGGGCSSYVGNTRRSGGQAVTLGWCHNRLGSIVHELGHSVGLWHEHTRADRDSYINVPSTSNNFRIQSTSDPRGTAYDFGSIMHYPMATRWSFGWVIMTMTTLGDTLYKSQNSPTIGQRVELSPLDIEGLLKLYPVIEAEEEVETTVTVTSVTATSVTVPQQPTLSCHYVSPGNPTDLKKKEWKCESHQEEYNVRCCSDATENPYPQGKWQQLSSNHVDHCPWVVSEALSEASEYKKCPKKMNYNDAFEYCANADARLCTREEMVHCSYGSGCGLGSKYMWTSSHDGPPTDNPTSAPTTVPPTLSPTTSSPTEAPSTSAPTQSPAAPIPTPTSSPTTFMPTSAPSPSTAQPTAKPTAEPQCHMVAPGRAKNLQKTPTKCAGHDETHRVRCCSDATVDPFPSGNWVLTNKNDCNNVWSAEKNMAAGEDGKCLSRMTYADASAYCTSVSARLCSLDEMVNDCTVLSDCKINKDHLWTSTPGGPPPPTTAAPTHAPTTPSPTTSPVIAPTKAPTPPTPQPMSAECHIVRTGKFSKSKPPICQDNDVKAKVVCCSDSDPASLSSQWKYNGASGVWSAGKDFASGSKSCRSGSTYDDADNFCSGLGTRLCTEQELEDNYATGTSCGYNSKYVWTSSTSV

>Diaphanoeca_grandis_m.134048/1728 g.134048 ORF g.134048 m.134048 type:complete len:729 (+) comp29716_c0_seq2:1462332(+)

MMLRCFVVAIIAVNYFAAAVDARPRAIREEEVDDVQIDIGVQRLPAGDHEGYDLLLTIDQFQNLHDVKIDSVEGRCLDKNGRVPFSIPKPGFTDSAEECLEWCVNINVMYQIEFGACEVYDTQDHDSFKCRALFANSLSNPLQTDTTTSNYVCFPLRQKNRRQRKSSSGLDSESRRALRDGNLWPDNTIPYTIVTSGSNSWGGNGKSQSQATNNVLSVIATYNQLTNVRFVPRTTQSRYVVIGYFGGGCSSYVGNVFAGQQITLGWCWNSIGSIIHEFAHAAGVYHEQSRRDRDDYLDVGVNASSNNNFDIQGNTDSRDLLYDFGSIMHYPLSTYWNPGYVIMTKTPLGEQRHDDQQAEIGTFAIGQRSVLSDLDIQGLALLYPPLASQSPTTSSPTPTPTTPAPTAQPTTSAPTIFVACHYASKGKKAVGSESCDDNDVLHPFRCCSDSVSQPSGNGWSQRSSGKDGGDCPWVASEKMGTGFTNCPGPKTWSEANQYCMDTGALNRLCTLDELQNECTLGSGCSITNTQMWTSTSNTVATSKPTPSPTTPSPTPSPIPSPTPSPTTPSPTPSPTLKSPTPSPTTPSPTPLPTTLSPTPSPTPSPGASCHYVSKGKNALGSESCEENDNSYPFRCCSDSNSEPDGDGWVRLSGKDGGDCPWAASRAMGVGFDDCPGSKTWSAANQYCMDTGTSNRLCTLNDLQNECTLGTGCSITKFKMWTGTSGGGT

>Diaphanoeca_grandis_m.211969/1460 g.211969 ORF g.211969 m.211969 type:complete len:461 () comp33115_c0_seq4:851467()

MLRFTVIASTLAFVALTTAAPAPRIHRDVSPDGGEGHDLLVTPDQFKKMRPGSLTPITGACAATKAASTAFVTTAECIDWCINTEIDMSKDTGACQYNTATGECTVFTAESFDGTTTASENTQCYSLLASDRRSKRGLVVDTLLWPNNEVIYEIVPSSDANSWATKLGGESHQTSALAYIQLAFDEYHTKTNVRFVPRTSQAHYIKIGYFGGGCSSHVGKNNFGAQPVTLGWCRNRLGSIVHELGHSAGLWHEHTRADRDQYLTVSDGANSNFNIARTDSRGIVYDFGSIMHYPLSTSWSGFVVTMTKTADGEALHTAQGNTQIGQRSGLSQLDIDGLAQMYPASDSPTPSPTPSPTPSPTPSPSPSPTQSPASSPPTVAPTECQITTVADHRYLPIDNVPGPAANRARTVMTTEECRARCRDTVGCAYFSRWNAGSGCHLSSSASVKDYRSGVTSGTAC

>Salpingoeca_dolichothecata_m.151057/1383 g.151057 ORF g.151057 m.151057 type:complete len:384 () comp24492_c1_seq1:7011852()

MLAPALLLLLLLGRLCRAIPTQTGNLNSNAELPYKVPGRHLPHAPPNVSLPAVPRSKRESTSPETETDFRLENPGLNLFEGDLKLKRRFIKRHYLRTDDDQTRKRRGVAKGLRFWPNAVVPYFFSSEVPSNTRAVVLKAMLWISSETCIQFVPRKSEREYLKIQSSSPGCFANMIGSLKNRWVYINLQPGGCSFTGIAAHELLHILGMWHEQSRADRDLHININYENIKVQANQFSKRNTDSLGFEYDFNSIMHYPLNAFAKQGTNSIAVKNTDLLARQEARRDWGGKIGQRLRLSDIDLGQLRAMYTCPTPTTTTTTTTSTTTTTTTTTTNHDLEFPVTTTTTTRSVLFPNETQHTTQQTHRIFPHDTNHAEPCLLNYVFVE

>Salpingoeca_dolichothecata_m.32380/1400 g.32380 ORF g.32380 m.32380 type:3prime_partial len:400 (+) comp14956_c0_seq2:1981400(+)

MLCVLLLALTTVLGFPNPRKYADSSRGWKSIADINVNADREFYNGDIVLTKKQKKELEHSRRDRRGLTSVTTLKWPNSIVYYIKDYSNDATLDRLIDDAIAQWEAQSCLQFVPRTNQNDYIRFRNNEEGCWSYIGRIGGAQTVHMERYGCYHLGIIAHEIGHAVGYHHEQSRTDRDTYVQINWQNIKDGYEFNFEKSSSTDSLGSPYDLRSIMHYGRTAFTSNGQNTLDVISNPYNVPLNQIGQIVGLTDIDGDQSRLLYPENCSPAPQPCSAVTLSNSPIGGTNGEYSQNGNINGRVSYVKGGYNIRWNSNSLVWEVRDGSSVELYSPTNVRAVHQASGTFYDDSTNRRDSSILMACSTAATTAGTTTGTTPDTTAGTTPGTTPGTTPGTTARTTSDTT

>Salpingoeca_dolichothecata_m.261240/11086 g.261240 ORF g.261240 m.261240 type:5prime_partial len:1087 (+) comp26781_c0_seq6:13261(+)

HTHTRTHMKLLLLLCAFSALLGAVTSKVQYKYYPIFRTYHEAQDACLADYGGTLVEITSQQIQPNIQTNIQALVPNSSEIIFIGLNDIIDEGQFRWATGPKVTYSNWANAKNGKEDCVTMDGNTGKWNIDKCDVRHHFVCQRNIEYKYIDSPSTWEDAQQFCVTTCGGTLAQPATPESQSAVLQAVSHNSKGAYIGLSDIEKEGKFIWGDGEAVEYSHWAPNSPNKANVKSDCVVMSKGGLWDDVDCSTKRPFVCQCPEFAVQYKHFPQAKTFAKAQESCEADFDGSLVEIISPSIARRIQYLVKGTPVYIGLTDFKFEAEFEWLSRDALAFENWAPGQPNNAGKEGEDCVITTTDGKWDDVHCEKISEYVCQKAPRAGGISSVDFANNTRDRRDTPAGKSACYIGHPPTRCNIGDVNDGVRCYPLCKGGYYGVGPVCWERCNPDYTDDGATCRRDNGYAKKSYGRGAGTIPKCCPSGYELNAGLCYKKCRSGYKGVAHICWQSSCPKHYRDYGALCHRPMDSHGKGFCCCGGCKSGYSNHGCICTRPPRTIRKSSYNRGVGKIPATFGAGACCGKDLNAGLCYNRCKTGYSGVGPVCWENCRSGFKNAGVFCSKLHIYGKKNYGRGAGYPASCKLSEKKGFLCESQCDILSKLPAIPEKRRRAIEEFGDDLPDDLAGGQKKRRGVFEGYKWPDAKFFYKWHSNVPSSMKGFFKDIFKVFESKTCLRFFENKPGDGASRVCEIKAYGSGRSKANLYGYTIKQVWSDMERSAMHEVGHALGLLHEQGHKSRDSYISDVKPDGNDNEISHYATTTAYDYQSIMHYSGFKVTNKAYFYQQARASSKDFVDTRFPSAGQRTFLSDGDVAILNDMHCGFEITVVVGTEAYAESRDTHYVGLVSSAGYCGEAGLPSLAKGSVRKVRIICANGGVPLYLRFRSGSNDAALIRHVSVRNSRGQLLLNQYTDQWVDGDGVPHQQKWYLRLAGLRPYGVVIRTSAGRHADSNGPHYFAMYAGIYLCRELKTINWAKGESRVIIFSCPGNPTELTARQGTKNGLLVESVTRRHRVQVIRKWLDADGPASMRYWSVRF

>Amoebidium_parasiticum_Apar_comp21710_c1_seq1m30654/1239

EQGGNKRPDDPYLWPDNTVIYKFSNEVKTRYPSMITEFQRALQIVSDATGGCIKSKPHTNEPHYIRIMKDPNPQVTCYSFVGYGSMWGVSDGNGYTYQDFNLYNTQYGDTCTDYIVVHELLHGIGVEHEHVRPDRDKYVIVYKNNIEPYFYETGYVKKDFTTFNLPFDFGSLMMYPYYSWAKPGKKYAMTKLDGSVWQNQESHLSKKDIDQMMALYKCASTTTTKTTTKTTTTTTKTTT

>Ministeria_vibrans_Mvib_g2222t1/16202

MKLLLVVTAVVMAVTGSAVSHGFRPGHVYSYQYSSQVVQSRSPRDAPHTGLKLACNTHFFVRGVHDTPDSIASDDPELIIALKIDQVKVSSPVEGTMYPPAVTEEYAKGLTQDVFHFTLQGGRVRQVWAAADEAVWATNIKRGIVSSLQLETPALLENGHSFASDETDTTGTCPTDHTVHRDDATDATASEHFVVEKTKDLAACRNRRVTHGHAARPERDEPMREALSSTYKGRYHFHTESGQLKRASIVETHFYDPYGSLASEKVHAHAQAQLTFIKARALASSKRSQASKNKHTSVRSVIDDTVAEDRRIAHTRTSLHVEDWHDTTVSDEAKLDLQRRTLPEQKRRLTSEERTTLLPRLLANVQLVHANPRGELSVFLASVTILKEHPRLMFHLRDLYFKPDGLFVDIIINMLVATGTDEGFDDVVDSVVDIPAFNNTHRNHFLLAVGLLDEPTPHMFDTIMRLVRSNKVRSGRYFEVGVNALLALGNMIRRHSASVSPTAPQASFIKDAAGILLTGLAEATLMKNSDTELLYVHALGNAGIGAAAPMLIARARDARHATMREAAIQAFRHIEPEHAPAQRADLITLALSQAHPKRDNERILALHTVLDTASHDELRTIAYHLRHERCENVRSYAYSRLRAISIDDPDMVRRRVTRDALDDFVSAGGIWDTSQSNLEQFVFTLGFSHNLEYFLVGGDKWGISVGWDLLDTATVTLQFAGSLRLNAVVQNRLFANAYAFSRRFRLLDVGFKINGLDWGTRLWSSRRRQVEEYDGTQLAMPNVPAARSAVKAFEALQEANHFGATQYERAVEEIARAGDQPNFSSYSPFGGKNKSPQESGESTRLESEPEMEQAHSRSRRGAGVHAGGRWENGIVYYHVNDNVDLDIHWANIQAGMDWYEANTCIRFYPRTTQGNFVEFKADEDADVCSSTLGTVGGGLGLQTVTLTSPSTCSVSTMIHELDHTIGTHHEQTRTDRDDFITVLWDNIADDKKHNYRKWNRFGAAGHNHGPYDYISNMHYSRFQASQQVFSTPNASQPLGGNVMTAGDVAHISFMYDESGICIDPPNRCNYIAVHGDTLFSIKSVLNDKFGMAITEEEIIERNKDTFPLIASLQFVTAGWELEVCDEGGTLEFKHKIEDAFNHPHVDMVDTNEFRLKLGKAPFENVTITISTTNNILHYPDYFYGPIVFNDRNWNIWQLIKMNVTGSGADTLSITGVGGGFDAAPALEIQFHGSVPGTCAPSIDNPLDNVNPPDPGSNSSIACECANGLDFQCSVKFCSVSIGHCSLINGLPNSGHIGSPVDGPDYWETCSVPAPSLPQSCTDFWNTIDWIPNGMRINMIHQCEAVLPVGVDPAIENMYNCIESELRSKMVDTSIFSGAYRASMRAAKLAAPEFDIEASEAYWASLLPSGSLDLSTKLIDIFDKAYGTCCCAQTPGQYVGWLTSLPREVPPELVNFNKIIHGVCEERTALNDRPVDLAGYVYANFVGGIYSYCYNIFGTYCDTGFDKSDPLGIGERRRSTTMSPSGQAAHAAATSMTPLSQLPKVDTDLVSPAGRHIKELYQSENVKVMARRSLIDWIDESGQVFECWSPDPVPHIAKLEFPFFSFTIPIPIFIGISLDLTVSAGAFLAFAATAETCPTSIEATIKVGPEAGVEVVASASINLFLIKGGVRLNLRLFDTNLWVNLHVSMAARPLSTCLSLTWECEALGGEVEVFVQIRNKIKWCKVWGVKLPCGLTWGSELSVPIPGTSFSVDAFDGERELWSNCEENGNFLPEVQTTSLPLTHEDISESGVFAYVDSNSDSVIWRVHANPKGKLSLLNPSTGYFRYKPSIHFSGQDKFYMIAYDGQEDGPPQEITVDVEGIPDAPRVEVEPAVGVEGNPARFVVNWDMVDRDESELLAFAICNCREENDTDHDEWGDACDNCPFVYNPTQGDIDGDGIGDACDSCNFTCTPPKNGVCCARLNETSYLYPLFPDPHFCFSDYSAEKCEDLETVHAGHFIVLQFHEGKQCDEVDCGVPHDAQCQVSADGLGCTVCPDKDKAERVFTLLSLIDKIKSKGRRIVALRDSIVALETERTRLDIRRAAAVEQLANADANPFFPPSDLADFKQDLADLIVDLEKSIRTREADIITLQEELDQCEKDLKGYLSDFVNSAKSPVCWPVKFKTSVNLVEECNCVDAPPFQCRPNAKRDGCVGFCDNKDVLTSAGVTTFGSSDSKMVNRTALETAIKEDEELLNSLGASAELLADTEAQLDAAIRFAPSQTHRDSYTLQLADAKTRRQEIEAEMTTLKGSLETMRRNLKVLIATTLECQPVRYAGSAVVECDCIEVQEPPTECEPTNDGLACTDVCSDTPDIPIPDKCEVRKISGVSDPAAGLYETDKYGQYCVGRCPNVPTKFCKPLFTNAVNTNERELKACGCAQNKCEKAIDPSKAGDLNNGYEMGYDIYGQYCKGECPDCNGGQCVGLFSNNGKDLKACECTTCDDSSSDSTTTTTTTSLTGSTTDIPLTTDFSSDSTASVLTGLNTNLDRLKALYGVLITQKCRPATYYEKTNLIKSCSCDGSDAPPRCQLTSSNSAMAGYFNKGYDDEGRRCVGSCPIDSTGQASLNCKGTWDYSTGKRLLTKCDCAQPPTCDVNFLDVVRTFTVSSSTNTDGTVTYAIGEACKQQVSKLDLTFIEGRELCQKRLGLSSLPATIKSYEVLGCGVTTPCGYGKTCLGSLSVRVCCQVPLKQLPRPTITIDRFELLTPSGGTFSAAPTIGEFMNFGKQLSFNVLIDRDRDRSKSPVSVSKRNLHVALVASTDSDLIPQMYGRLDEDVCLSCKGLLKSKTFKVVSGGSMSVDIEYSSSRNKYTQAGSLPSNTRLDFGDVSSTSCKKDTRFLGLVELYNDENGQLWATRLFDSAGNLATSKFAYESQGCANAPSVRRRSTNEELFHVGAALPLAAFLPDSLLFNAVRVSRQPAYDNVPEHWSLLSGRPIVSQAEVRAADDEYERMVAEERERRSSSSYTPPVTPLSQAFPKGVCGCLGLQLPTNYTLYKRGVAMDPLEAEYITPEDMPFIDIQGDSDDWGRFGLMIKGITMEKDVPTKAAVTMRPVGVVIDPLNDAPYFTTASLPASATVVSGSHFDFPGEMINDVDAFEGDLVVTVASKSGRGRVWLNGQAPSTVHVYHIGEALHIDDEVFGEQVVFDDVSTFSAHDEIQGIPVDGNGGQYLRIRGSLVDVNETMQMGFRYEALPGPAGNDTLMMSIDDTFFSLLPRGARGYVSTSFPITITASAATNTPPAWTWSNGLVFVASHSTVALTGLSVVETDGDALTVHVNVTANAPVQSISLSNASFTSSTSLVLECSNATACNSALAQFSVTLSSEASCPVFVHLSVSEGSAAPVTLVVIVQPQQLPALLAASTMAASSSSSSSSLPCNSTAPVCGATETRSDIVHAEDLTHHVSCPAVYSGACPCWFGKRLVFNAQKVYANGSEYSGATGQALFDGGLLFFSGNVNNDNPNRWDAFHMTVYCNASSSSLSSVAADLYQHTSSSSGTPRMKALFTRSGPKCMLLSLRTDSANGTECSLPTTMDDSNEVQHYLLCTEDADSTLSTCTDGDVTQAVYPSTDVASPMNVCPQTHIGTCPCWMSDNVPQTMELLYANGTVIPDRKSFVKFTGRIAIVSGGLDTTNAGNQLTLMFTTQCNTNDSTATTVEGTFDFRLTSNPEISASRLPFVVTRSAADIADDCFTLNLRIDTGNDTVNNCTVPETVPLSGAPVPAANGGSAFLFGRICRNRAPCNFVPPCNDTPAPVCGATETRSDIVHAEDLTHHVSCPAVYSGACPCWFGKRLVFNAQKVYANGSEYSGATGQALFDGGLLFFSGNVNNDNPNRWDAFHMTVYCNASSSSLSSVAADLYQHTSSSSGTPRMKALFTRSGPKCMLLSLRTDSANGTECSLPTTMDDSNEVQHYLLCTEDADSTLSTCTDGDVTQAVYPSTDVASPMNVCPQTHIGTCPCWMSDNVPQTMELLYANGTVIPDRKSFVKFTGRIAIVSGGLDTTNAGNQLTLMFTTQCNTNDSTATTVEGTFDFRLTSNPEISASRLPFVVTRSAADIADDCFTLNLRIDTGNDTVNNCTVPETVPLSGAPVPAANGGSAFLFGPPAVPLPPSAPEPCLPCYSASTSEPCTPCCATTNATMLSGTTNTAEDTAVNVTLAFANPTASLADVYTVHITCPDGAMTLLADATAAGISVERQQAWPLVGTANLATDTVLRGPVSGLVTVLNRVMYTPARDVAGITRLKFDFYGASEDLSAAAQLYDYAHLEHRLWVSPVSDPPRLEIPGIVFVDETTETQLWALMAADVDDNAEEQIDLLTVTVTSVRNTFHINKPTYLAIAFTVGDGTSASTMTFLARPEGATAALNEIYYEAGGCVQEGCSSGVDELAITLTDQAGLTVTKKIFIDVVCKAEPPLLVTDDVTGDEDTNITLSAAQVTLRDPEEFLTVFLTPQEGSAPFSLTHGTLQGITWQVLPSDLNAGVINVILEEDNHDNIVFDVVAYTLEPSNGRVARNTSTFTVNVLPVNDPPQVIMPAQQKGKEDIWFALDPILFYDIDVPSGTGYNFRAELSVNDGTLSYYNHSTPMCTSGLTCTGLYTSSVSFEGPHEAVNGMLGRIFYKSDHNWNGIDVLSCTIRDMGSVGSGPVGVAVGTVKVTVEPVNDAPYFSVTLVPAQNGLCLPDYGNKTCLPRIHDFSPYEDHPVALPVSNPIQLDDVDSKPTDFIYLRISAGRGSLSMPAISSTTLTFTTGDGTDDQLIVLNGTLSEIQTLLSLLTYTPLADMNGDDYIEVYARDGPSDGTQPMDTSHQEGVYGFYVHPVNDPPFVSMTMTPTMVRPGQLSIGSVSIFDDATEELSNNDVIRVTLKVFNGLIRISHVNAPVSIVSGSQNSASVTIQGPLDSVVSDLQSIEYRPDAFLDGSDFDHSVDSLVVTVDDLGHFGGCPSPLSQTPCNQRTRQNHTIIVEECTGTLSWTTFPVGTGSIDGQDTWNYADLPPNFFDEFDADGDVVLAEDETTLVFDEPPPPNPTISYESTVFHDVIIPATDPYETEYLVPFRVNAITPFGVPGGTMCAETAPAVIGEDISSVSDLLMRTSFNVGYGVIGVNVTVRANQPVYVYFNEEYFDVHTHTGGDCSTESSFVMPFWFAPDGATREDDTFRNTLHLRAPRRPDFIEARVSVLSCSGQRPDCLKHKCRSDVYETLYSTAGIEGTTNTGICPRGCVCGAKEMCIDNSCGNGIVETFNGEECDDGNFVNDDACSNTCKKGVDRSCHIEQYNTDELRKEMGNSSLTAEQRARIQLIIDKVDAGTAVGTVDGFDKYGRYCAGACLDDPTQPPCLALWTNDDTELKDCGCVPSGSSGGGTGCRIEVNPYFQYSGDVDSTIFDRKGRYCTGSCATDATKQCLAKWSNDNQDITACDCPPQQTGCRVNDKRNGCEGACPADKSEYNLSLLYGGQQLKQCLPRFVTYGMVRVLSRCDCGSTDTTTGCRVITDKLTGAKSCGGACPTSGNKCQKKVFRGSTYCDCVPDFTTPKYGCRVTAADECVGVCKNTLLPCKEVRQGGKMKSCGCGIIDLGCTVNFDAQTRTPLSCTGTLTGSSTSCAALTQTPVGIVECRGDDAAVDPAKGCYFNRVLGECSGRCFDSNNVARECQAVTKGVFTIGCVCPKPESTSMSTEPCQIINDKCVTIPNLGSCTGFTAGKFKCDAVYNRLGQLTACNCPTPAERTCSTTDPTSLCFIKPQQQGSLKGIASLKPSTSRLLSKLESAGKAVDKVVNVTFSLDVPVDVPQDVSFVVQQDVLPSCATLADDTTTECTNSHSEDIADPTEARAFDPLTLAFTITAVNDSDSSFQRGILFTSPLVVSASVPAAECDAETLSSVRLYYFDVDCFGDTGVAADCWKDAGATCPADQAMHDVTGCTLTVHVCHLTAFSLGVGSTTLSTGASIAIVAGAAAAAIVVLLLVLLFVVRRRRSQQQQQQQQQKGVKEGRGSGGGLAYSQAHLVTRGAQESNDEQVAMQGSKSMTQVELTRAIGAEMGSQPPPMLLVDDPESRRQFTNDVDDFHSFADDSQSFNDDSRSFADSDIGVSEQERERRRKARMQRKQSRLENIDMPSWLSDQLSAREVRHSKRGTQPGEVVYDRDGNAVETSAHGSHQLDVEPTAGERLGMEQSPVSAPDGSSVSDGVLPKGAL

The extended zinc-binding sequences are highlighted in blue for reference.
